# A Review of the Research Progress of Sensor Monitoring Technology in Harsh Engineering Environments

**DOI:** 10.3390/s25206308

**Published:** 2025-10-12

**Authors:** Qiang Liu, Yang Wang, Fengjiao Zhao, Chuanxing Zheng, Jinping Xie

**Affiliations:** 1 School of Ocean Energy, Tianjin University of Technology, Tianjin 300384, China; hustlq2003@email.tjut.edu.cn (Q.L.); 13731262850@163.com (Y.W.); zhaofj2007@126.com (F.Z.); 2 Tianjin Huashui Engineering Consulting Co., Ltd., Tianjin 300204, China; 3 Bei Fang Investigation Design & Research Co., Ltd., Tianjin 300222, China; xie_jp@bidr.com.cn

**Keywords:** harsh environment, sensor technology, monitoring, data transmission

## Abstract

**Highlights:**

Reviews recent advances in mechanical, optical, and acoustic sensing technologies for complex engineering environments such as high temperature, high pressure, and corrosive conditions.

**What are the main findings?**

- Sensor monitoring technologies have made significant progress in adapting to harsh engineering environments, including underground, underwater, and high-risk industrial settings.
- Integrated early warning platforms combining sensor networks and communication systems have enhanced real-time monitoring, remote control, and disaster prevention capabilities.

**What is the implication of the main finding?**

- The application of advanced sensor systems improves safety, operational efficiency, and decision-making accuracy in engineering projects.

**Abstract:**

With the continuous growth in the demand for safety assurance in major projects and monitoring in extreme environments, sensor technology is facing challenges in harsh working conditions such as high temperatures, high pressures, and complex liquid media. This article focuses on typical complex environments such as underground and marine environments, systematically reviewing the basic principles, performance characteristics and the latest application progress of mechanical, optical and acoustic sensors in complex environments, and deeply analyzing their applicable boundaries and technical bottlenecks. The transmission mechanism of sensor data and the system architecture of the engineering monitoring and early warning platform were further explored, and their key roles in real-time perception and intelligent decision-making were evaluated. Finally, the core challenges and development opportunities currently faced by complex environmental sensing systems are summarized, and the future development directions, such as multi-parameter fusion, autonomous perception and edge intelligence, are prospected. This paper aims to provide a systematic theoretical basis and engineering practice reference for the design of sensors and the construction of monitoring systems in extreme environments.

## 1. Introduction

Against the backdrop of increasing intelligence in urban infrastructure, energy development, ecological protection and other fields, high-timeliness and high-resolution environmental and structural state data have become the key foundation for decision support. As the core tool for obtaining this crucial data, sensor systems are becoming increasingly prominent in modern engineering and environmental monitoring [1,2]. Wearable devices in daily life can monitor physiological signals such as body temperature [3], pulse [4,5], and blood pressure [6]. In smart cities, sensors are relied upon to monitor water pollution [7,8], noise pollution [9], and air pollution [10]. In engineering environments, monitoring the health of structures relies on self-sensing concrete sensors [11] and measurement robots [12]. The diverse application demands are constantly driving the development of sensing technology towards higher precision, stronger adaptability and greater intelligence.

With the in-depth application of multi-field intelligent systems, the stable operation ability of sensors in complex environments has become the core bottleneck restricting the perception ability of systems and has become a key research direction in current sensor technology. Many scholars have made outstanding contributions to enhancing the practicality of sensors [13]. A buoy platform for marine environment monitoring was designed, integrating various micro-sensors such as capacitive silicon-based accelerometers, gyroscopes and magnetometers for real-time monitoring of sea surface waves. In addition, there are buoy devices applied to the observation of key marine parameters such as salinity [14], temperature [15,16], wind speed [17,18] and precipitation [19]. Water pollution and marine debris can be monitored by optical fiber microfluidic sensors [20], magnetic sensors [21] and sonar devices [22]. The changes in the status of organisms can be effectively and promptly observed remotely by means of chlorophyll and cyanobacteria sensors [23,24] and aptamer sensors [25]. Due to the particularity of the tunnel structure, it is necessary to rely on distributed optical fiber sensors [26,27,28] to conduct real-time settlement monitoring and structural health monitoring of the tunnel, and promptly detect and eliminate dangers. The health and lifespan of geotechnical structures are monitored through optical fiber sensors [29,30] and tilt sensors [31]. In the field of oil and gas wells, it is impossible to directly observe the underground environmental conditions. The pressure and temperature data underground can be monitored through sensor ball monitoring equipment [32] and optical fiber Fabry–Perot optical fiber pressure sensors [33]. Similarly, in underground mines, sensor monitoring equipment is also needed to monitor the temperature, air, pressure and other underground environmental conditions. Ensure the safety of staff [34,35].

Environmental monitoring using the sensing characteristics of pressure-sensitive elements [36], optical fibers [37] and acoustic waves [38] in harsh environments has become a common method. The so-called harsh environment [39,40,41] typically includes extremely high pressure [42], high temperature [43,44], strong corrosion [42,45,46], and severe mechanical vibration [47] among other harsh conditions. These factors usually lead to a decrease in the sensitivity of sensor devices [48], an increase in signal drift [49], and a shortened service life. Furthermore, the challenges not only exist at the sensor body end but also extend to the data transmission link. For instance, under strong electromagnetic interference [50,51], monitoring data may be distorted or even lost [52], which will further weaken the stability of the monitoring system. Therefore, the entire process from sensor monitoring equipment to data transmission links is confronted with numerous challenges, which are also a key factor restricting the long-term stable operation of sensor monitoring systems in harsh environments.

In recent years, in response to the monitoring demands of underground high-temperature, high-pressure, and complex liquid environments, many scholars have carried out relevant research from multiple perspectives. For instance, Kusumi Anjana et al. [53] systematically reviewed the application principle and deployment method of optical fiber sensors in geological disaster monitoring. A.S. Fiorillo et al. [54] summarized the material configuration and sensing mechanism of the novel piezoresistive sensor in high-pressure environments. Washim Reza Ali et al. [55] explored the failure modes and applicable scenarios of acoustic sensors composed of piezoelectric materials in extreme environments. Limu Qin et al. [56] analyzed the performance differences in hydrophones in marine observation and biomedical fields, and compared the adaptability of different calibration methods. Yaqin Wu et al. explored the real-time perception and analysis of various physical parameters in the underground mining environment based on the intelligent monitoring platform [57]. The above research has summarized in-depth the application situations of sensors in various fields. However, most of them have focused on the application of a single type of sensor (such as pressure sensors, optical fiber sensors, and acoustic wave sensors), and there are relatively few systematic reviews on key links such as communication transmission, post-processing, and visualization of monitoring data [58,59,60]. In fact, Based on a thorough understanding of the functions of mechanical, optical and acoustic sensors, improving their data transmission links and intelligent visualization processing procedures is of great theoretical significance for building an efficient and intelligent monitoring system [61,62].

**Figure 1:**
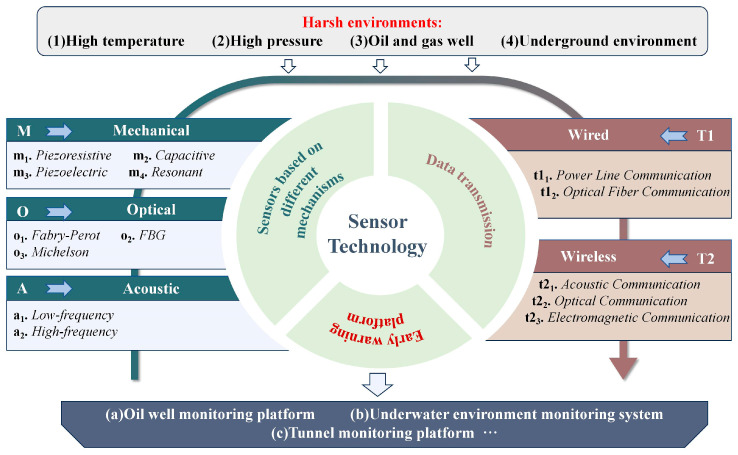
Sensor monitoring technology under different mechanisms in harsh environments.

Therefore, this paper reviews the key technologies and application progress of mechanical, optical and acoustic sensors in underground high-temperature, high-pressure and complex liquid environments, and conducts in-depth discussions on data communication methods and visualization technologies. Firstly, the core working mechanisms and typical application scenarios of the three types of sensors are summarized, and their applicable working conditions, performance advantages, and technical limitations are further analyzed. Secondly, the current communication methods applicable to this type of environment were summarized, with a focus on reviewing their transmission stability and environmental adaptability. Meanwhile, the architecture and feedback mechanism of the sensor data early warning platform were discussed, and its key role in achieving real-time monitoring and assisting decision-making was clarified. Finally, this paper summarizes the main challenges and development opportunities that complex environment monitoring equipment faces in special scenarios, such as underground and underwater, and looks forward to future development trends such as multi-parameter fusion, autonomous perception, and edge intelligence. This paper aims to provide systematic references for the design of sensors, monitoring data processing and intelligent visualization suitable for underground high-temperature, high-pressure and complex liquid environments, and to promote the engineering application and cross-expansion of such technologies in intelligent monitoring of extreme environments. As shown in Figure 1, the types of sensors and data transmission technologies applicable to the sensor monitoring system in harsh environments are demonstrated.

This article makes the following specific contributions, addressing gaps in existing reviews (for further detailed information, please check tables in Appendix A):

1. It provides a comprehensive synthesis of the most recent progress on sensor monitoring components tailored to underground high-temperature, high-pressure, and complex liquid environments, which have not been systematically reviewed in prior work.
2. It updates previous reviews by providing the latest analysis of mechanical, optical, and acoustic sensors, including their principles, features, limitations, and application scenarios.
3. It summarizes the state-of-the-art in communication and transmission methods for monitoring data under harsh subsurface environments, an area that is often underrepresented in the existing literature.
4. It expands the discussion to integrated monitoring and early warning platforms, highlighting how sensor technologies and communication systems can be linked for practical engineering applications—an integration rarely addressed in comparable reviews.

## 2. Materials and Methods

To ensure the reliability and comprehensiveness of the dataset, a systematic search was conducted across multiple scientific databases, including Scopus, IEEE Xplore, and Web of Science. The search period was restricted to publications between 2016 and 2025. A combination of keywords was employed, such as “harsh environment sensor”, “sensor communication”, “underwater sensor monitoring”, “downhole sensor monitoring for oil and gas”, “high-temperature and high-pressure sensor monitoring technology”, “sensor monitoring platform”, “mechanical sensors”, “optical sensors” and “acoustic sensors”. Boolean logic operators were applied to integrate these terms and refine the retrieval process.

**Figure 2:**
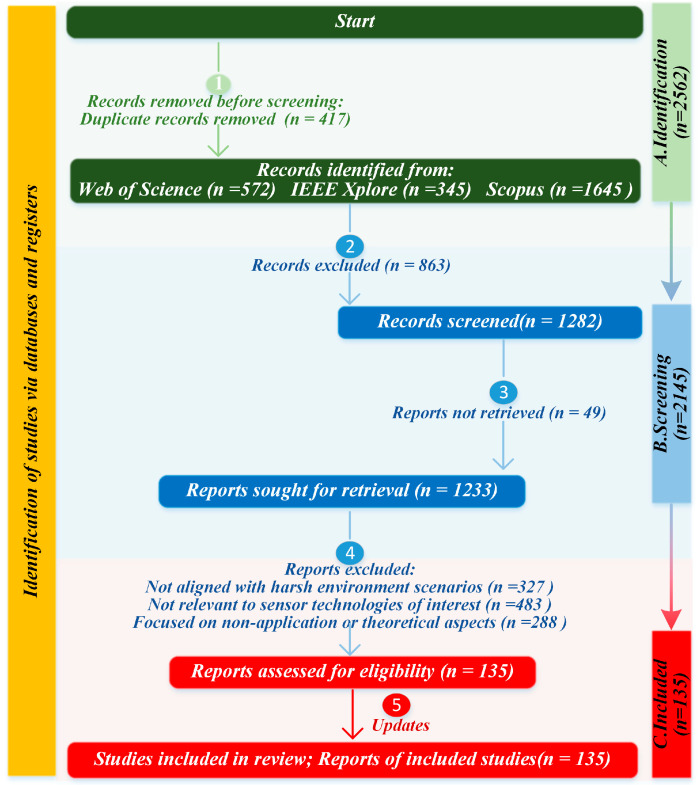
PRISMA flow diagram.

During the literature screening process, duplicate records were removed first. Subsequently, a preliminary screening was conducted based on titles and keywords to exclude studies unrelated to engineering environmental monitoring or limited to materials theory. Finally, the full texts were reviewed, and literature meeting the following criteria was retained: sensor technologies applicable to high-temperature, high-pressure, oil and gas wells, underwater environments, and electromagnetic interference environments; wired and wireless technologies for sensor data transmission; and early warning platform systems based on sensor technology. The PRISMA flowchart illustrating the literature filtering process is shown below, as presented in Figure 2.

## 3. Analysis of the Principles and Applications of Sensors Under Different Mechanisms

Sensors, based on their material properties or structural design, can respond to external physical stimuli, thereby achieving functions of data acquisition, processing and feedback. According to the differences in the perception mechanisms and energy conversion methods of external stimuli by sensors, common sensors are generally classified into three categories: pressure sensors [63], optical sensors [64], and acoustic wave sensors [65]. Pressure sensors complete signal transduction through changes in electrical characteristics such as resistance [66], capacitance [67], charge [68] or frequency [69] caused by mechanical stress, and are suitable for directly measuring pressure or strain changes in the environment. Optical sensors rely on changes in light intensity [70], wavelength, phase [71] or polarization caused by external disturbances, and have advantages such as strong anti-electromagnetic interference ability and long-distance transmission capability. Acoustic wave sensors detect the changes in the characteristics of acoustic waves propagating in the medium (such as amplitude, frequency, and propagation time), and are widely used in structural defect identification, liquid medium detection, and complex medium state recognition [72,73]. Therefore, this study starts from the working mechanisms of these three typical sensors, systematically sorts out their application adaptability and key performance in extreme underground environments, and explores the performance differences and development potential of them in high-temperature, high-pressure and liquid media.

### 3.1. Mechanical Sensors

In engineering environmental monitoring, pressure serves as a key physical parameter reflecting environmental variations. It is widely used to assess operational conditions and safety risks under complex working environments, and holds significant research and practical value [74,75]. Mechanical sensors convert sensed pressure into electrical or mechanical signals through various transduction mechanisms—such as piezoresistive, capacitive, piezoelectric, or resonant effects—enabling efficient acquisition and transmission of pressure information. Owing to their structural adaptability, high measurement precision, and strong resistance to interference, Force Sensors exhibit broad application prospects and distinct advantages in fields such as underwater exploration, downhole operation monitoring, underground structural safety assessment, and high-temperature equipment operation monitoring.

#### 3.1.1. Mechanical Sensors Based on Different Response Mechanisms

In pressure monitoring, sensors typically rely on measurable changes in electrical parameters—such as resistance, capacitance, or voltage—in response to external stress, thereby achieving high-sensitivity environmental detection. Based on their signal transduction mechanisms, pressure sensors are generally classified into four types: resistive, capacitive, piezoelectric, and resonant.

- Piezoresistive Sensors

**Figure 3:**
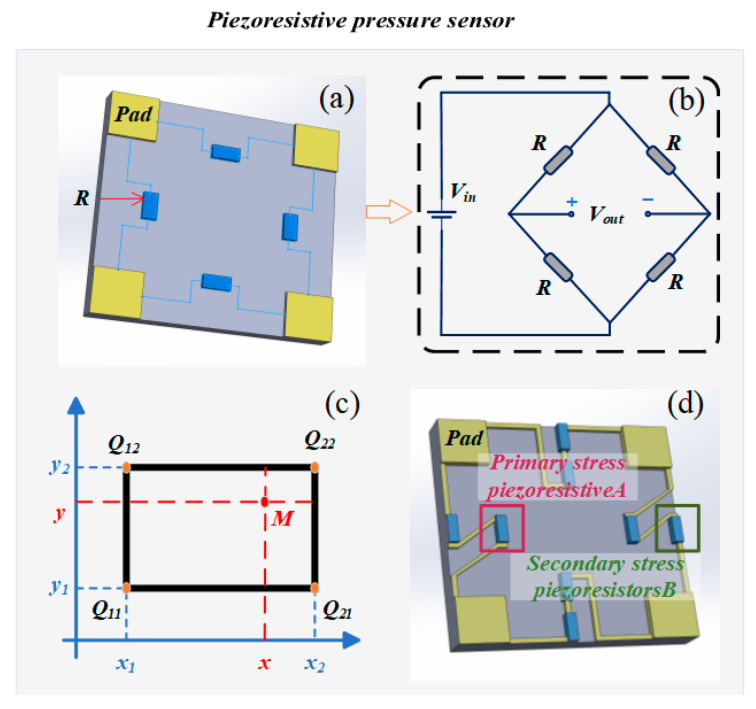
Piezoresistive principle and optimization methods. (**a**) Piezoresistive schematic diagram [81]. (**b**) Wheatstone Bridge [81]. (**c**) Bilinear interpolation method [76]. (**d**) Diaphragm secondary stress utilization methods [79].

Piezoresistive pressure sensors operate based on the resistive effect of materials, where external pressure induces changes in the bulk or contact resistance of the piezoresistive material, enabling pressure signal detection and conversion. As shown in Figure 3a, in a typical piezoresistive sensor under applied force, the contact area and current path of the material change, resulting in resistance variation. When integrated with a Wheatstone bridge circuit (Figure 3b), this variation is converted into a voltage signal, enabling quantitative measurement. These sensors feature a simple structure, fast response, and high sensitivity, and are widely adopted as mainstream solutions in engineering monitoring applications. In practical applications, the electrical signals generated by piezoresistive sensors can be transmitted through power line communication to achieve data transmission, which can ensure reliable data transmission while avoiding additional wiring.

In complex monitoring scenarios—particularly in high-temperature and high-pressure underground environments—piezoresistive sensors are often the preferred choice due to their miniaturized design, good integrability, and cost-effectiveness. Among them, silicon-based piezoresistive sensors are widely applied in typical settings such as underground oil and gas wells, geotechnical structures, and tunnel deformation monitoring, owing to their excellent linear output, high resolution, and stability. However, high-temperature conditions can significantly alter the resistivity of piezoresistive materials, compromising the accuracy and long-term reliability of the sensors. To address this issue, various temperature compensation and performance optimization methods have been proposed. To mitigate errors caused by thermal sensitivity, numerous studies have explored algorithmic compensation, material design, and structural optimization, significantly improving sensor performance under extreme conditions. For instance, one study proposed a temperature compensation method based on bilinear interpolation (Figure 3c), integrating least-squares fitting and interpolation algorithms, which reduced the full-scale error from 21% to 0.1% [76]. Wenbin Su et al. integrated a platinum resistance thermometer and a thermal compensation circuit, reducing linearity error from 8.12% to 0.06% full scale (F.S.) within the range of 0–40 MPa and 0–80 °C [77]. Additionally, the improved gray wolf optimization (IGWO) algorithm has been applied to optimize temperature compensation models. By tuning the nonlinear convergence factor and weight parameters, the approach achieved full-scale errors below 0.03% and local errors below 0.02% [78]. In terms of structural optimization (Figure 3d), the DSSUM (Double Stress Utilization Method) significantly improved sensor sensitivity to 32.35 mV/kPa—a 50.32% increase—while maintaining excellent linearity (0.031% F.S.) [79]. For sapphire-based silicon sensors, which suffer from accuracy degradation in high-temperature environments, a high-temperature compensation system has been developed. This system maintained measurement accuracy better than 1‰ F.S. from −20 °C to 140 °C, and better than 3‰ F.S. from 140 °C to 250 °C, effectively mitigating thermal drift [80]. In summary, multidimensional temperature compensation and algorithmic optimization have become key research directions for enhancing piezoresistive sensor performance, laying a solid foundation for their stable operation in high-temperature, high-pressure, and complex liquid environments.

- Capacitive Sensors

**Figure 4:**
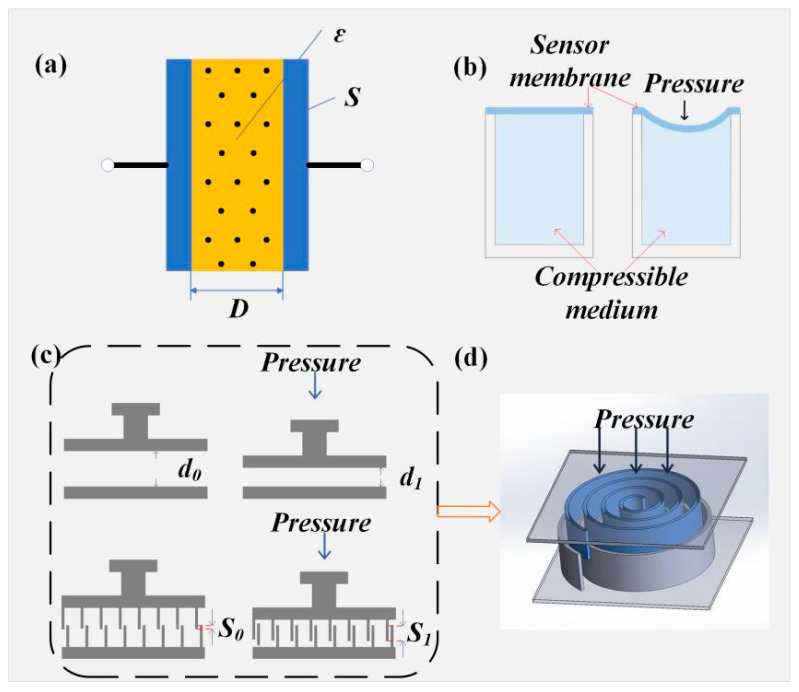
Capacitive principle and optimization methods. (**a**) Principle of capacitance. (**b**) Compressible dielectric capacitor [84]. (**c**) Force variations in typical capacitors and linear comb capacitors. (**d**) Force variation in spiral comb-shaped capacitors [85].

Capacitive pressure sensors mainly comprise two conductive electrodes separated by a dielectric layer (Figure 4a). When external pressure is applied, the gap between the electrodes changes, leading to variations in capacitance, thereby enabling pressure detection. This capacitance change, induced by pressure-driven geometric or dielectric property variations, constitutes the fundamental electromechanical transduction mechanism. Besides changes in electrode spacing, the sensor’s capacitance can also be modulated by altering the electrode overlap area or substituting different dielectric materials. Various structural designs result in diverse capacitive response modes. Due to its electrical output characteristics, it can be easily combined with electromagnetic communication systems and optical fiber communication systems, ensuring low-loss and anti-interference data transmission in underwater and high-temperature environments.

Capacitive pressure sensors exhibit high sensitivity and excellent dynamic response, along with robust stability under high-temperature and underwater complex environments, making them a key technology for pressure monitoring in extreme conditions. Current research mainly focuses on high-temperature modifications of sensor core materials and structural designs. Silicon carbide (SiC), as a third-generation semiconductor material, demonstrates superior performance compared to traditional silicon (Si) in high-temperature and high-pressure sensor applications due to its wide bandgap, high thermal conductivity, strong radiation resistance, and corrosion resistance. Quanwei Zhang et al. systematically reviewed the application potential of SiC-based capacitive pressure sensors in extreme environments, confirming their advantages in thermal stability and mechanical strength [82]. Based on these findings, a novel SiC capacitive pressure sensor featuring a vacuum cavity and cylindrical interlayer structure was developed, significantly enhancing capacitance output sensitivity and linearity. Neglecting temperature effects, the sensor achieved a maximum sensitivity of 1.34 fF/MPa with a nonlinearity of 0.094% full scale (FS); at an operating temperature of 20 °C and bonding temperature of 70 °C, sensitivity increased to 1.25 fF/MPa and nonlinearity decreased to 0.089% FS, corresponding to improvements of 6.72% and 5.32%, respectively [83]. The response performance of capacitive sensors depends not only on material properties but also closely on their geometric structures. For example, structures based on dielectric elastomer membrane bending and compressible medium volume changes (Figure 4b) enable synergistic control of sensitivity and deformation capacity, accommodating diverse pressure loading modes [84]. Additionally, Wenjie Chen et al. proposed a novel capacitive pressure sensor with a spiral comb-shaped electrode structure (Figure 4c,d), where capacitance changes are primarily induced by variations in electrode overlap area rather than the electrode gap changes typical of conventional designs, significantly improving sensor linearity and directional adaptability. Experiments demonstrated that under horizontal and vertical orientations, this structure’s sensitivity and linearity improved by approximately 0.13–0.33% and 0.06–1.10%, respectively, compared to traditional straight comb structures [85]. Thus, through synergistic optimization of materials and structures, capacitive pressure sensors can maintain high accuracy and stability in underground high-temperature, high-pressure, and complex liquid environments, providing reliable support for engineering monitoring under extreme conditions.

- Piezoelectric Sensors

**Figure 5:**
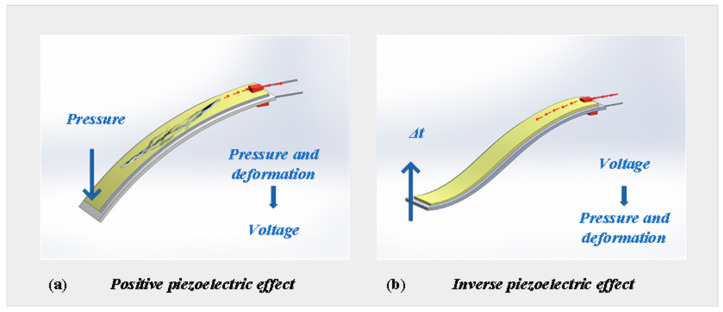
Piezoelectric effect. [90].

Piezoelectric pressure sensors operate based on the piezoelectric effect, which converts externally applied mechanical pressure into electrical signals. Figure 5a,b illustrate the direct and inverse piezoelectric mechanisms, respectively. For example, in the widely used piezoelectric polymer PVDF (polyvinylidene fluoride), external mechanical stress causes deformation of its internal polarization structure, leading to charge separation on the surface and the generation of an electric potential. Once the stress is removed, the charges dissipate and the piezoelectric effect vanishes. By monitoring the generated voltage, the applied pressure can be indirectly inferred. In addition to PVDF, commonly used piezoelectric materials include inorganic compounds such as GaN (gallium nitride), ZnO (zinc oxide), and PZT (lead zirconate titanate), which offer superior thermal stability and charge output characteristics, making them suitable for harsh operating conditions.

Piezoelectric pressure sensors are particularly well-suited for monitoring transient impacts and dynamic pressure environments, such as underwater explosions, structural impact tests, and blast wave propagation. Their fast response time and self-powered operation make them highly applicable in these scenarios. Dongbao Wang et al. developed a custom PVDF-based piezoelectric sensor, which was tested in underwater explosion experiments to verify its response performance. However, due to PVDF’s high temperature sensitivity, thermal shocks (e.g., rapid temperature shifts from 290 K to 354 K) significantly affect its measurement stability [86]. To mitigate the adverse effects of thermal gradients, Tim Krause proposed applying a 1 mm thick RTV silicone protective layer on the sensor diaphragm, which effectively buffers thermal shock transmission. Experimental results showed that the maximum measurement error of unprotected sensors reached 16%, while those with silicone protection maintained errors below 6%, significantly improving environmental adaptability and measurement stability [87]. Furthermore, the introduction of novel piezoelectric materials offers improved solutions for high-temperature and high-pressure monitoring. For instance, GaN thin-film-based piezoelectric pressure sensors exhibit stable voltage responses over a temperature range of 100 °C to 350 °C. The correlation coefficient (R^2^) between pressure and output signal exceeds 0.98, indicating excellent linearity and repeatability, suitable for pressure monitoring in extreme environments [88]. Beyond conventional pressure monitoring, piezoelectric sensors are also widely used for structural dynamic response monitoring and natural frequency extraction in multi-physics coupling scenarios. Shaojun Du et al. designed a piezoelectric sensor device for extracting the dynamic deformation response of cantilever beams. Their findings demonstrated that the device can capture natural frequency information of components during wind tunnel testing, highlighting its engineering value in seismic monitoring of building structures and structural health assessment in aerospace applications [89]. In summary, piezoelectric pressure sensors have become a key technology in dynamic pressure monitoring, shock wave detection, and structural vibration analysis, owing to their high-speed response, passive operation, and strong adaptability to dynamic conditions.

- Resonant Sensors

**Figure 6:**
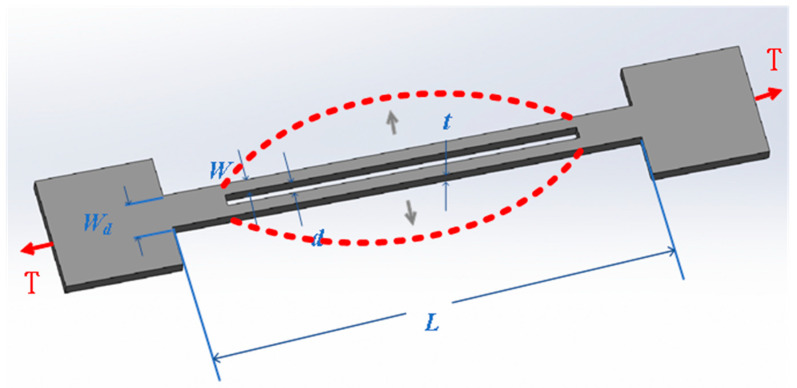
Schematic diagram of DETF structure, red dashed lines indicate vibrations extending to both sides [97].

Resonant pressure sensors are based on the sensitivity of the resonant frequency to the stress state and indirectly measure external pressure by tracking variations in the natural frequency of resonant elements. As shown in Figure 6, when external pressure is applied to the sensor’s sensitive unit, it will cause a change in the internal stress field of the resonant structure, which in turn leads to a shift in the resonant frequency. This frequency change can be precisely mapped to the corresponding pressure value. According to the material differences used in their resonant units, resonant pressure sensors can be classified into two types: silicon-based and quartz. Silicon-based resonators usually rely on mature MEMS processes for miniaturization manufacturing, featuring low cost and high integration advantages. However, they have insufficient thermal stability at high temperatures, and their excitation and reading structures are relatively complex. In contrast, quartz-based resonators have excellent frequency stability and high Q value characteristics, making them suitable for high-precision scenarios. However, their manufacturing process is complex, the cost is relatively high, and they have strict requirements for processing technology control.

Although resonant pressure sensors have attracted widespread attention due to their outstanding accuracy and long-term stability, their complex structure and cost issues still limit their large-scale application in engineering practice. To meet the ultra-high-pressure measurement requirements in extreme environments, Quanwei Zhang et al. developed a high-precision sensor system based on a double-ended tuning fork (DETF) quartz resonator. Its structure consists of a pressure cylinder and a lever forming the primary force transmission unit, and the resonant output module is composed of two DETFs in a differential structure. The experimental results show that the linear error of this sensor is 0.0112%, the hysteresis error is 0.0142%, the repeatability error is 0.0317%, and the total accuracy reaches 0.0365%, which can meet the high-precision measurement requirements in harsh environments [91]. The optimization of structural design is also an important direction for enhancing performance. For instance, the adoption of an embedded boss stress isolation structure can effectively shield the interference of packaging stress and temperature stress on sensitive chips, demonstrating excellent measurement repeatability under low static pressure and low hysteresis conditions [92]. In addition, Thanh Tuong Pham et al. designed a resonant sensor based on AT-cut quartz resonators and adopted a temperature compensation method based on Beat Frequency analysis. The core principle is that the beat frequency is composed of the difference between three times the fundamental frequency and the third overtone frequency, which has a linear relationship with temperature but is insensitive to pressure changes, and can achieve temperature drift compensation [93]. However, this method has only been verified within the range of 30–60 °C and 0–350 kPa, and there are still problems with the limited coverage of temperature and pressure. To expand the adaptability of the sensor under extreme temperature and pressure conditions, researchers have proposed a typical laminated all-quartz resonant structure. This structure can simultaneously excite fundamental mode and overtone frequencies, achieving decoupled measurement of pressure and temperature. Within the test range of −40 °C to 150 °C and 0 to 120 MPa, this structure achieved a full-scale error of ±0.015%, demonstrating extremely high precision and stability [94,95]. In addition, the introduction of new piezoelectric materials has also injected new impetus into the development of resonant sensors. For instance, by using Langasite (LGS) instead of traditional quartz as the piezoelectric material to construct a dual-mode resonator, not only the high-temperature stability is enhanced, but also the ability to simultaneously measure pressure and temperature and achieve temperature compensation is achieved. Relevant experiments have verified the feasibility and engineering potential of this scheme [96]. Its development direction will focus on the development of high-performance resonant materials, low-cost structural optimization, and multi-physics field compensation mechanisms with a wider range of coverage.

#### 3.1.2. Analysis of Mechanical Sensor Applications

As shown in Table 1 below, the latest applications of mechanical sensors with different operating mechanisms in high-temperature, high-pressure, oil and gas wells, and underwater environments are summarized. The table also highlights their differences in temperature range, pressure tolerance, applicability to specific environments, and main functions.

When silicon piezoresistive pressure sensors are used for high-temperature and high-pressure underground monitoring, their measurement accuracy is significantly influenced by ambient temperature [76]. At normal temperature or in scenarios with low precision requirements, the influence of temperature on measurement errors can be ignored. However, in long-term high-precision monitoring scenarios, high-temperature calibration or the construction of temperature compensation models are required to enhance stability and reliability. However, the expensive equipment required for calibration and the high labor cost have limited its wide application. To cope with high-temperature environments, the new ceramic-based thin film strain gauge can operate stably at up to 700 °C without depositing an oxide layer, providing an effective means for in situ static and dynamic strain monitoring at extreme temperatures. In addition, thanks to the excellent hydrophobicity of PDMS materials, piezoresistive sensors demonstrate good stability in damp and underwater environments, providing technical support for underwater wearable devices.

Capacitive pressure sensors can enhance their sensitivity by adjusting the volume of the compressed medium and the structure of the cavity, making them suitable for air, shallow water and deep water environments. However, under high-pressure conditions, changes in mechanical properties may still affect its measurement accuracy [84]. In addition to its application in deep-water environments, it can also be used for pressure measurement in high-temperature conditions. In the research [100], the high-temperature capacitive sensor has a relatively high operating temperature and demonstrated excellent performance within the range of 0–350 °C. According to the monitoring characteristics of capacitive pressure sensors, in addition to pressure monitoring, liquid level monitoring can also be carried out. This type of capacitive pressure liquid level sensor is feasible. The conductivity of water may affect the output signal, which becomes a key issue for further improvement [74].

Piezoelectric pressure sensors have significant advantages in dynamic pressure measurement due to their ability to directly convert mechanical energy into electrical signals, especially demonstrating high responsiveness in extreme environments such as underwater explosion tests. However, thermal shock can adversely affect the performance of piezoelectric materials, such as PVDF. The adverse effects brought by sudden temperature changes can be alleviated by switching to new materials or coatings (such as RTV silicone), reducing the impact of thermal shock effects and enhancing their dynamic stability.

The resonant pressure sensor indirectly measures pressure by detecting changes in resonant frequency. It adopts a quartz resonant unit with a double-ended tuning fork (DETF) differential structure, which can achieve high-precision and high-stability pressure output, and is particularly suitable for application scenarios with ultra-high pressure and high reliability requirements [91]. This type of sensor can also simultaneously measure temperature, expanding the functions of this type of sensor. Although resonant pressure sensors have significant advantages in performance, their complex processing and high manufacturing costs have become important factors restricting their large-scale application. Therefore, optimizing structural design, improving processing techniques and selecting multiple material options to reduce costs will be the focus of future research.

The research reviewed in this section shows that:

1. The adoption of temperature compensation algorithms and temperature compensation devices can effectively enhance linearity, sensitivity and reduce errors.
2. Based on the characteristics of capacitors, various forms of improvements can be made. In the future, improvements can be made in multiple aspects, such as sensor manufacturing materials and dielectric materials, to meet the monitoring requirements of different scenarios.
3. The application of new material coatings can effectively reduce the impact of thermal shock on piezoelectric pressure sensors.
4. By adopting a more stable and efficient new pressure conversion structure, the accuracy of the original parameters can be effectively enhanced and the error of the original parameters can be reduced.
5. The temperature compensation system based on beat frequency analysis can significantly improve the measurement accuracy of resonant pressure sensors.

### 3.2. Optical Sensors

Optical fiber sensors perceive and respond to physical parameters such as pressure, temperature and strain by monitoring the changes in their internal optical properties (such as refractive index, interference phase, reflection intensity, etc.) under the influence of external environments. When disturbed by external pressure or temperature, the propagation path, phase or reflection frequency of light in the fiber core will change, and thus can be captured by the demodulator and converted into the corresponding environmental information signal. With significant advantages such as high resolution, excellent anti-electromagnetic interference capability, high temperature resistance, corrosion resistance, and long-term stability, optical fiber sensors are particularly suitable for long-term deployment in complex and harsh environments. Its compact size and light weight also enable it to be embedded in narrow Spaces in extreme scenarios, and it has the ability to perceive both pressure and temperature parameters, providing highly competitive solutions for engineering applications such as underground monitoring, structural health assessment, underwater system perception, and high-temperature scene monitoring [105,106]. With the continuous development of optical fiber preparation technology and optical microstructure design, the application potential of such sensors in multiple fields is constantly expanding [107,108,109].

#### 3.2.1. Optical Sensors Based on Different Response Mechanisms

According to the differences in sensing mechanisms and optical structures, common optical fiber sensors can be classified into the following three major categories: Fabry–Perot cavity sensors, fiber Bragg grating sensors, and Michelson interference sensors.

- Fabry–Perot Sensors

**Figure 7:**
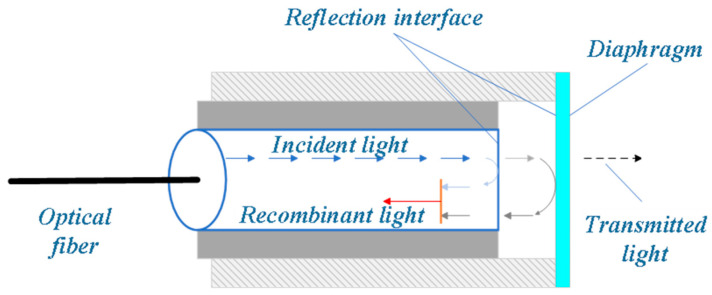
Schematic diagram of the Fabry–Perot style [119].

The Fabry–Perot optical fiber sensor achieves high-precision measurement based on the optical path variation in the interference cavity structure. Its basic structure is shown in Figure 7. A typical FP cavity can be divided into two types: intrinsic and non-intrinsic. The intrinsic type forms an interference cavity by directly constructing two highly reflective surfaces inside the end face of the optical fiber. For the non-intrinsic type, a micro-gap is set between two sections of optical fiber, and the reflective surface is jointly formed by their end faces [110,111]. When external pressure or temperature acts on the FP cavity, it will cause changes in the cavity length or refractive index, which in turn leads to a shift in the interference spectrum. By analyzing the spectral response of the interference signal, the inversion measurement of external physical quantities such as pressure, temperature and concentration can be achieved [112,113].

FP optical fiber sensors are widely used in complex environmental monitoring scenarios such as marine, underground and high-temperature environments due to their high sensitivity, strong resistance to electromagnetic interference, and adaptability to remote distributed measurement. However, under conditions such as seawater corrosion, high-temperature expansion and medium disturbance, the sensor is prone to performance degradation. To address this issue, Guoqiang Li et al. proposed a novel corrosion-resistant FP optical fiber sensor for marine environments, which was used for temperature and salinity monitoring, successfully enhancing the detection accuracy of temperature (maximum resolution 1.15 × 10^−4^ °C) and salinity (maximum resolution 1.35 × 10^−4^ PSU) [114]. In addition, to achieve multi-parameter decoupling, researchers have developed multi-cavity structured optical fiber sensors. For instance, the cascaded dual FPI system utilizes the thermal and optical differences between the two cavities and demodulates the interference transmission valley through the transmission matrix method, thereby achieving dual-parameter measurement of bicarbonate concentration and temperature [115]. Another design of a parallel dual FP interferometer based on the cursor effect also demonstrated high-sensitivity monitoring capabilities and long-term stability for temperature and pressure [116]. Material matching and process reliability under high-temperature working conditions have become the key factors restricting the practical application of FP optical fiber sensors. To address the impact of high-temperature thermal expansion and contraction on packaging stability and interference accuracy, researchers adopted a fully silica structure without adhesive design to avoid device failure caused by mismatched thermal expansion coefficients of materials. For instance, the all-quartz structure optical fiber FP pressure sensor developed by Jiashun Li et al. maintains good stability within the range of 23–800 °C, with a pressure sensitivity of 3.54 μm/MPa [117]. In terms of manufacturing processes, micro-electromechanical systems (MEMS) technology makes it possible for sensors to be miniaturized and mass-produced. The MEMS FP sensor, combined with wavelength tracking technology, covers a measurement range of 0–10 MPa, with a pressure resolution of 0.017% and a nonlinear error of only 1.44%. The high consistency and repeatability of the new MEMS FPI process provide a technical foundation for its engineering monitoring deployment in harsh scenarios such as oil and gas wells and petrochemical pipelines [118]. Fabry–Perot optical fiber sensors, with their flexible structural design, adjustable material systems and integrated manufacturing processes, have become a key optical sensing solution for monitoring underground high-temperature, high-pressure and complex liquid environments. These innovative designs and advancements in manufacturing processes provide reliable technical support for the application of FP-type optical fiber sensors in complex environments. It is worth noting that optical fibers themselves possess dual attributes of sensing and communication. This enables Fabry–Perot optical sensors not only to achieve high-precision monitoring but also to be integrated into optical fiber communication networks to realize long-distance sensing monitoring and real-time data transmission.

- Fiber Bragg Grating Sensors

**Figure 8:**
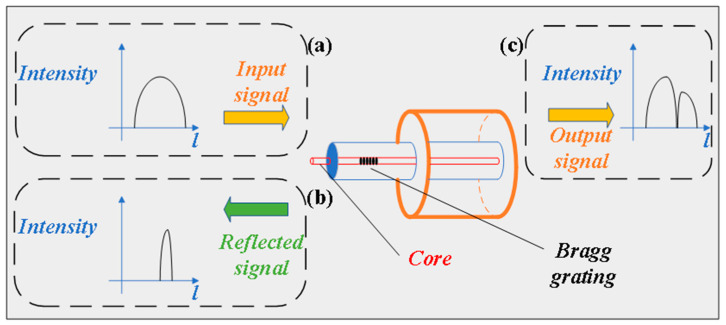
Schematic diagram of fiber Bragg grating. (**a**) Signal input. (**b**) Signal reflection. (**c**) Output of reflected signal [29].

Fiber Bragg grating (FBG) sensors achieve physical parameter perception by utilizing the periodic refractive index modulation structure in optical fibers. When broadband light that satisfies the Bragg condition is incident on the grating region, narrowband light that meets specific conditions will be reflected in the grating region, as shown in Figure 8. The Bragg condition is satisfied by the following Equation (1):

(1) $$\lambda_{B}=2n_{eff}\Lambda$$

Among them, $\lambda_{B}$ is the central reflection wavelength of FBG, $n_{eff}$ is the effective refractive index coefficient of the core, and Λ is the inherent period of the grating.

When external physical quantities (such as temperature, pressure, etc.) act on FBG, it will cause changes in $n_{eff}$ and Λ, which in turn leads to a shift in the reflected wavelength. By monitoring this wavelength drift, the corresponding changes in physical quantities can be inversely deduced, achieving non-electrical contact and highly sensitive physical quantity monitoring.

FBG sensors have been widely studied and applied in multiple extreme environments such as marine engineering [120], oil logging [121,122], and aerospace [123]. Especially in high-temperature and high-pressure environments such as oil and gas wells, traditional sensor structures face challenges such as large temperature drift and low sensitivity. For this reason, researchers have proposed a variety of structural optimization schemes. For instance, a composite structure sensor that combines a diaphragm, an irregular-shaped bracket and dual FBGS can simultaneously monitor temperature and pressure, and is suitable for working environments ranging from 50 °C to 200 °C and from 0 to 40 MPa. This scheme achieved a temperature sensitivity of 31.8 pm/°C (R^2^ = 0.9997) and a pressure sensitivity of 50.6 pm/MPa (linearity 0.21%), demonstrating excellent adaptability to high temperature and high pressure [124]. The dual FBG sensor with a hinged rod enhanced structure significantly improves pressure sensitivity to 5.227 pm/kPa, making it suitable for scenarios with high requirements for low pressure and high resolution, such as oil and gas pipelines [125,126]. The square diaphragm design, in combination with the vertical arrangement of FBG, effectively reduces non-uniform strain interference, achieving a sensitivity of 3.402 pm/kPa and a linearity of 0.9985 within the range of 0–200 kPa. However, the suspended grating structure may cause stability issues [127]. To solve this problem, Mingyao Liu et al. further optimized the diaphragm structure and proposed a regional strain homogenization design, enabling the FBG to be directly attached to the uniform strain area and achieving higher stability and sensing accuracy. The sensitivity of this design is 30.8174 pm/MPa within the range of 0–30 MPa, and the linearity reaches 0.9996. It is particularly suitable for harsh working conditions such as oil and gas wells [128]. In complex liquid environments, FBG sensors also demonstrate excellent adaptability. For instance, an FBG liquid pressure sensor based on the deformation difference between a metal housing and a polymer achieves signal transduction through the pressure coupling deformation of the housing. This sensor can integrate multiple FBG nodes of different wavelengths, support multi-point series measurement, and significantly expand the measurement range and sensitivity. Within the range of 0–15.5 MPa, the pressure sensitivities of the two series FBGS reached 51.296 pm/MPa and 46.117 pm/MPa, respectively, and the linearity was 0.9970 and 0.9981, respectively. They are suitable for complex liquid monitoring scenarios such as deep marine water [129]. Through multi-mode optimization of structure, material and grating arrangement, FBG sensors can achieve high-sensitivity and high-stability pressure and temperature monitoring in complex high-temperature and high-pressure liquid environments, providing technical support for the engineering application of such sensors. In addition, as FBG relies on the waveguide characteristics of the optical fiber itself, its sensing signal can be directly transmitted and multiplexed over long distances through the optical fiber communication link.

- Michelson Sensors

**Figure 9:**
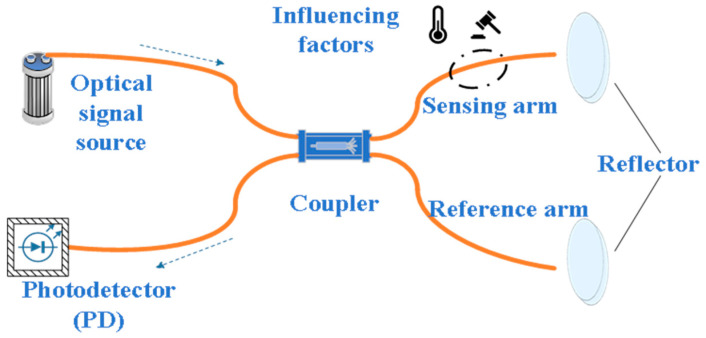
Schematic diagram of Michelson style [134].

Michelson optical fiber sensors are based on the principle of Michelson interferometers and achieve precise perception of external physical quantities (such as temperature, pressure, humidity, etc.) by monitoring the changes in interference fringes. As shown in Figure 9, the incident light is divided into two beams, the reference arm and the sensing arm, by the optical fiber coupler. After propagation, respectively, they are reflected and re-superimposed to form interference. When external physical quantities act on the sensing arm, the optical path changes, causing a phase shift in the interference signal. The interference light intensity can be expressed by the following Equation (2):

(2) $$I=I_{1}+I_{2}+2\sqrt{I_{1}I_{2}}\cos\left(\Delta \Phi \right)$$

Here, *I* represents the interference light intensity, *I*_1_ and *I*_2_ respectively represent the light intensity from the reference arm and the sensing arm, and ΔΦ represents the phase difference between the two beams of light, which satisfies Equation (3):

(3) $$\Delta \Phi =\frac{2\pi \cdot \Delta L}{\lambda }$$

Here, λ represents the wavelength of light and is related to the optical path difference ΔL.

Michelson sensors have demonstrated significant advantages in high-temperature monitoring, especially in fields such as oil and gas wells, metallurgical processing, and aerospace. Research shows that by adopting special optical fiber materials and microstructure design, its temperature adaptability and measurement accuracy can be effectively enhanced. The temperature sensor constructed with germanium-doped optical fiber has an inner cladding of pure silica. The existence of this internal cladding structure reduces the number of interacting cladding modes. The temperature sensitivity is 48 pm/°C in the 20–250 °C temperature range, increases to 78 pm/°C in the 250–600 °C range, and there is no obvious thermal hysteresis effect below 600 °C. It shows good high-temperature stability [130]. Jiahao Guo designed a conical optical fiber structure with gold film reflection enhancement, which was constructed at the end of the SMF to enhance the end reflection rate. Experiments have proved that its temperature sensitivity is 80 pm/°C in the range of 100–450 °C, and it increases to 109 pm/°C in the range of 450–900 °C. It still maintains excellent linearity and stability at high temperatures [131]. Michelson interferometers are also widely used in the measurement of environmental parameters such as humidity and salinity, demonstrating excellent adaptability. In humidity monitoring, the perception of 55–85%RH (relative humidity) is achieved by modulating the intensity and wavelength of interference light, with maximum sensitivities reaching 0.224 dB/%RH and 0.133 nm/%RH, respectively, meeting the precise measurement requirements in medium and low humidity environments [132]. In marine salinity measurement, to address the challenges brought by deep-sea high pressure, high salt corrosion and temperature disturbances, researchers have successfully reduced the influence of environmental disturbances on interference fringes through the symmetrical arrangement design of the sensing arm and the reference arm. When tested in a high-pressure chamber simulating the deep sea at 115 MPa, this optical salinity sensor achieved a salinity resolution better than 0.0003 PSU, demonstrating outstanding performance across the entire deep-sea environment [133]. Michelson optical fiber sensors, with their sensitive and stable interference detection mechanism and scalable structural form, demonstrate wide adaptability in high-temperature, high-pressure, highly corrosive and multi-physical coupling environments.

#### 3.2.2. Analysis of Optical Sensor Applications

As shown in Table 2 below, the latest applications of optical sensors with different mechanisms in high-temperature, high-pressure, oil and gas wells, underwater, and underground environments are presented. The table also highlights their differences in temperature range, pressure tolerance, applicability to specific environments and main functions.

The FP sensor with a fully silica structure can operate stably at temperatures up to 800 °C and features excellent thermal stability and structural compatibility. The manufacturing process of this type of sensor is relatively mature and suitable for mass production, thus demonstrating great application potential in the industrial field [117]. In addition to pressure measurement, FP sensors can also be used for high-resolution measurement of seawater salinity, relying on their high-reflectivity interference cavity structure. It should be noted that this type of sensor is prone to being affected by factors such as bubbles, turbulence and mirror condensation water droplets in practical applications, which interfere with the cavity reflection characteristics and thereby reduce its signal-to-noise ratio and measurement stability. Therefore, its robustness needs to be further enhanced in terms of structural optimization and environmental compensation strategies.

FBG sensors have been widely used in pressure and temperature monitoring in high-temperature and high-pressure environments such as oil and gas wells due to their excellent anti-electromagnetic interference capability [124,125]. This type of sensor senses external stress or temperature changes through variations in the reflected wavelength, featuring high sensitivity and fast response speed. However, temperature has a significant impact on its reflection wavelength, which can easily lead to cross-coupling between pressure signals and temperature signals, affecting the monitoring accuracy. Therefore, systematic research needs to be carried out from aspects such as grating writing process, cladding material selection, protective housing design and signal decoupling algorithm to enhance its stability and reliability in complex environments [128,129].

Michelson optical fiber sensors achieve parameter perception based on the phase difference changes in the interference arm and are suitable for high-temperature monitoring scenarios. Research shows that after adopting a special optical fiber structure with an internal cladding, it has good signal stability and noise resistance below 600 °C, but its stability drops significantly above 700 °C [130]. To enhance its high-temperature performance, Jiahao Guo et al. introduced a conical structure and coated the end of the optical fiber with a gold film, which increased the reflectivity and enabled it to maintain good sensitivity and reliability in an environment as high as 900 °C [131]. In addition, in the field of salinity monitoring, fiber-optic sensors have better long-term stability compared to traditional multi-electrode conductivity sensors due to their susceptibility to electrochemical drift [133]. However, the high-pressure environment in the deep sea may affect the stability of the micro-cavity of its optical structure, leading to a decrease in measurement accuracy. In-depth research on high-pressure immunity is still needed.

The research reviewed in this section shows the following:

1. The sensitivity, measurement range and stability of multiple sensors can be improved through various combinations such as cascading and series connection.
2. Improving the shape of the diaphragm can enhance the performance of the sensor. An appropriate diaphragm shape can effectively reduce the impact of non-uniform strain.
3. Improving the structure at the end of optical fibers can enhance the reflectivity.
4. Sensors are made of new high-temperature and high-pressure resistant materials to enhance reliability in high-temperature and high-pressure environments.

### 3.3. Acoustic Sensors

Acoustic sensors play a crucial role in numerous complex environmental monitoring scenarios. This type of sensor receives or excites sound waves and analyzes the characteristic changes during their propagation (such as frequency, amplitude, propagation speed, etc.) to obtain external environmental parameters. Acoustic signals can usually be converted into electrical or optical signals to achieve subsequent processing and analysis [135]. Acoustic sensors are widely used in multiple engineering fields such as underwater acoustic detection, environmental noise monitoring, liquid level measurement, marine structure sound field assessment, and underwater communication. They particularly demonstrate extensive applicability in oil and gas exploration, marine engineering, and underwater robot systems.

#### 3.3.1. Acoustic Sensors Classified by Frequency Bands

**Figure 10:**
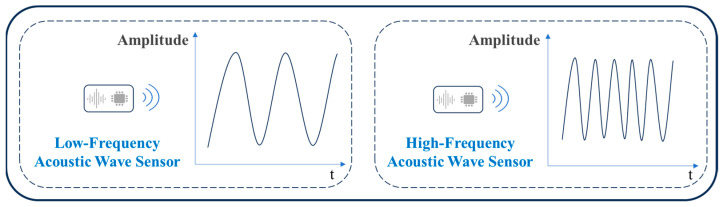
Low-frequency acoustic sensors and high-frequency acoustic sensors [136].

As shown in Figure 10, acoustic sensors can be roughly classified into low-frequency acoustic sensors (generally below 500 Hz) and high-frequency acoustic sensors (generally above 20 kHz) based on the different working frequencies of sound waves.

- Low-frequency Acoustic Sensors

Low-frequency acoustic sensors play a significant role in environmental monitoring fields such as earthquake monitoring, low-frequency noise detection in the ocean, and early warning of volcanic activities due to their superior long-distance transmission capabilities and penetration. In recent years, numerous studies have focused on enhancing the performance and integration of low-frequency acoustic sensors in extreme environments. For instance, researchers have developed a fiber optic hydrophone for seismic monitoring in volcanic areas by winding optical fibers around a flexible axis to sense weak strain. This sensor achieved a sensitivity of 350 nm/Pa within the range of 1–80 Hz. The average background noise measured on the seabed was 100 μPa/$\sqrt{HZ}$, and the correlation coefficient with the reference hydrophone (PZT hydrophone) in the same frequency band was as high as 85%, verifying its effectiveness and stability in the detection of submarine volcanic and seismic sound fields [137]. Another study constructed a low-frequency acoustic interference sensor based on silicon nitride (Si_3_N_4_) film and the Fabry–Perot interference principle. Within the range of 1–250 Hz, its frequency response exhibits excellent flatness, with a sound pressure sensitivity of up to −152 dB (re 1 rad/μPa) and a fluctuation amplitude of less than 0.8 dB. This acoustic sensor demonstrates outstanding performance in ultra-low frequency sound field measurement [138]. Although fiber-optic low-frequency acoustic sensors have advantages such as high sensitivity and resistance to electromagnetic interference, their complex structure, high manufacturing cost and strict requirements for light sources limit their large-scale application. Therefore, in recent years, the introduction of water gel materials in low-frequency underwater acoustic sensors has attracted widespread attention [139]. Researchers have designed a novel capacitive hydrophone based on hydrogel design, which has a higher sensitivity compared with traditional hydrophones. The key lies in the addition of micro-nano structure electrolyte layers. The collaborative concentration of CNT (carbon nanotube) network topology and micro-pyramid array structure enables the hydrophone to achieve a high sensitivity level within the range of 20 Hz to 125 Hz. It achieves a sensitivity level of −159.7 dB, which is approximately 33.29 dB higher than that of the commercial hydrophone (RHC-14). Moreover, this hydrophone also demonstrates excellent SNR (signal-to-noise ratio), capable of detecting weak acoustic signals as low as 0.3 Pa [140]. By integrating MEMS technology with piezoelectric ceramic tubes, the sensitivity of acoustic detection can be enhanced. The vector path adopts a lightweight dual-pillar ciliary structure, which optimizes the balance between natural frequency and sensitivity, while the scalar path further improves detection performance through a series of interconnected piezoelectric ceramic tubes. This hydrophone exhibits excellent performance in the frequency range of 20–500 Hz; at 500 Hz, the vector path sensitivity reaches −170.5 dB and −170.8 dB, whereas the scalar path sensitivity is −168.5 dB [141]. Inspired by sensory mechanisms in nature, Xuyu Zhang et al. proposed a resonant fiber-optic acoustic sensor based on a bionic ciliary-coupling structure, achieving a sensitivity of −118.0 dB (re 1 rad/μPa) at 54.0 Hz [142]. Low-frequency acoustic sensors, with their excellent long-range detection capabilities and robustness in complex working conditions, have demonstrated great development prospects in fields such as environmental monitoring, underwater engineering, and geological disaster early warning. Currently, the research trend is gradually evolving towards high sensitivity, high integration, and high environmental adaptability. In addition, the inherent long-distance and low-frequency characteristics of these sensors make them particularly suitable for acoustic communication and optical fiber communication.

- High-frequency Acoustic Sensors

High-frequency acoustic sensors typically refer to acoustic wave sensors with operating frequencies higher than 20 kHz. Due to their high-frequency characteristics, they possess excellent spatial resolution and beam control capabilities, and are widely used in underwater detection, high-temperature environment monitoring, ultrasonic imaging, and structural health diagnosis, among other fields. In recent years, researchers have been continuously enhancing the performance and environmental adaptability of high-frequency acoustic sensors by focusing on two major directions: structural optimization and material modification. In terms of structural design, the fiber-optic high-frequency acoustic sensor significantly enhances its sensitivity through a dual-resonant cavity structure. One cavity is used for mechanical amplification of the sound pressure signal, while the other serves as a functionalized Fabry–Perrow (FP) optical cavity. The thickness of the sensing film and the gap between the two cavities are precisely regulated. At a frequency of 100 kHz, this sensor achieved a sensitivity of 26.9 μPa/$\sqrt{HZ}$, which is far superior to that of traditional single-membrane FP acoustic wave sensors [143]. For piezoelectric acoustic wave sensors, the structure of piezoelectric micro-mechanical ultrasonic transducers (PMUT) has also been significantly optimized. For instance, by thickening the bottom electrode layer of the PMUT to make it serve both as an electrode and a support structure, multiple PMUT units are connected in parallel to form a two-dimensional array. This not only enhances the acoustic output capacity but also effectively reduces the total impedance of the system [144]. Furthermore, through the integrated structural design of PMUT, the transmitter and receiver can be placed in the same transduction cavity, achieving simultaneous transmission and reception mode operation, providing a new idea for the development of portable underwater ultrasonic imaging equipment [145]. To enhance the response performance of PMUT during the transmission and reception phases, Sara Mansouri et al. proposed a novel design using a C-slot structure. This structure helps to reduce the stiffness of the diaphragm, enhance the amplitude, and guide the formation of a piston-like vibration behavior. Compared with the traditional boundary clamping structure, the quality factor of this design is increased by 122.5% to 13,069. At the resonant frequency of 98.02 kHz, its displacement sensitivity and voltage sensitivity are, respectively, increased to 929.22 nm/V and −106.88 dB, which are 7.6 times and 12.69 times higher, respectively. It can be used in the fields of sonar and hydrophones [146]. In terms of material improvement, high-frequency acoustic wave sensors put forward higher requirements for high-temperature-resistant piezoelectric materials. To adapt to harsh environments, researchers have developed a Nb^5+^ donor-doped BiScO_3_-PbTiO_3_ ceramic material. The ultrasonic transducer composed of this material exhibits excellent piezoelectric performance below 400 °C, and this piezoelectric material has great application prospects in the field of high-temperature ultrasonic applications [147]. Similarly to low-frequency acoustic sensors, high-frequency acoustic sensors can also be employed for damage detection. Haoming Huang applied a micro-knot resonator (MKR) with ultrafine fibers in an ultrasonic underwater detection device, encapsulated with a double-layer PDMS film. The sensor exhibits an average acoustic sensitivity of −177 dB in the 180 kHz–1 MHz range, demonstrating the application of photoacoustic detection technology for broadband high-frequency signal detection underwater [148]. A novel ultrasonic waveguide sensor, designed on the principle of wave-mode conversion, is capable of exciting horizontal shear waves within the waveguide plate. Owing to the plate’s intrinsic thermal insulation and dissipation properties, this sensor enables reliable monitoring of pipeline thickness at elevated temperatures of up to 500 °C. Furthermore, the innovative structure allows the acoustic source to overcome the conventional limitation of shear waves by converting them into longitudinal waves, thereby enabling the use of a standard longitudinal-wave probe. This design not only enhances measurement flexibility but also significantly reduces production costs [149]. These studies have improved the performance of high-frequency acoustic wave sensors in different ways. Through continuous breakthroughs in structural optimization and material selection, the monitoring capability and signal processing performance of high-frequency acoustic sensors in extreme environments have been significantly enhanced, which can effectively improve their application adaptability. The high spatial resolution and fast response of high-frequency acoustic sensors are particularly suitable for optical fiber, acoustic, and optical communication, achieving accurate, high-speed data acquisition underwater.

#### 3.3.2. Analysis of Acoustic Sensor Applications

As shown in Table 3 below, the latest applications of acoustic sensors with different frequency ranges in high-temperature, high-pressure, subsurface infrastructure, and underwater environments are presented. The table also highlights their differences in applicable environments, frequency range, and main functions.

Due to the low-frequency characteristics, low-frequency acoustic wave sensors are widely used in the field of environmental monitoring and have a good application foundation in earthquake and marine monitoring. The strong penetrating power and long-distance propagation ability of low-frequency sound waves make them widely used in fields such as seismic wave detection, volcanic activity early warning and deep-sea noise measurement [137]. Low-frequency sensors are mainly evolving towards high stability and high integration. Combined with a new optical structure, the ability to identify weak disturbances can be effectively enhanced.

Significant progress has been made in the material improvement and structural optimization of high-frequency acoustic sensors. High-frequency sensors feature excellent spatial resolution and beam directivity, and have unique advantages in complex working conditions such as underwater imaging [145] and structural health diagnosis [147,152]. By integrating innovative designs such as new materials and new structural designs, the sensitivity, quality factor and integration degree of sensor equipment have been effectively enhanced, enabling the sensor to maintain high-precision response in complex environments such as high pressure, high temperature and strong interference.

The research reviewed in this section shows the following:

1. Hydrogel materials have good application potential in the field of underwater acoustic sensors and can significantly improve sensitivity.
2. Low-frequency acoustic wave sensors have broad prospects in environmental detection, such as seismic wave detection, volcanic activity detection and underwater noise measurement.
3. By integrating the typical structures of traditional acoustic sensors with mechanical and optical sensors, the detection performance of acoustic sensors can be significantly enhanced.
4. Enhance the performance of PMUT during the transmission and reception phases by leveraging the C-shaped slot design. In addition to the C-slot design, various new structural designs can also be developed to enhance the performance during the transceiver stage.

### 3.4. Cross-Category Sensor Performance

After analyzing the characteristics of various sensors (Table 4), a cross-category comparison of mechanical, optical, and acoustic sensors is presented. The comparison covers mechanism, sensitivity, operating range and frequency, anti-electromagnetic interference, stability, packaging, and cost, providing a reference for selecting suitable sensors in different harsh environments.

## 4. Transmission Methods for Monitoring Systems

**Figure 11:**
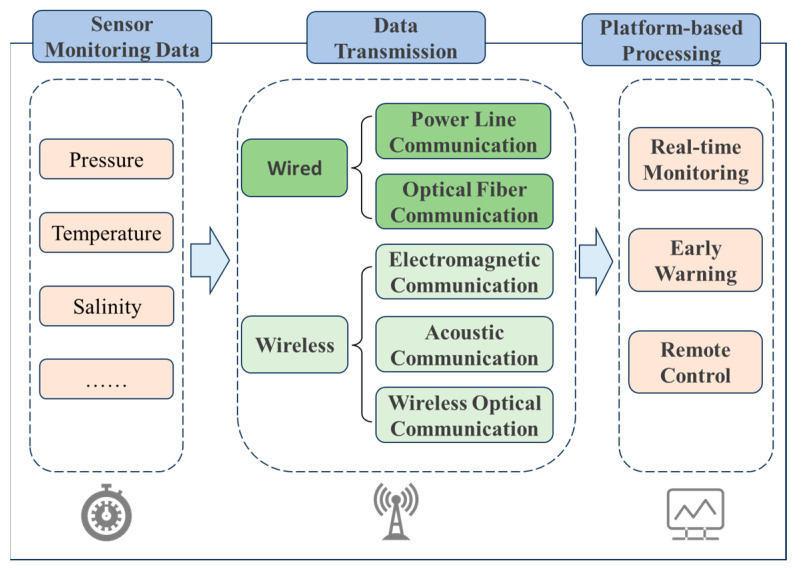
Flowchart of data monitoring, transmission and processing.

In various complex environments, after sensors complete on-site data collection, how to stably, efficiently and losslessly transmit the collected data to the early warning platform has become one of the key links in building a complete monitoring system [157]. Meanwhile, to achieve intuitive display and intelligent analysis of the monitoring results, the transmitted data needs to be further connected to the early warning platform to realize real-time observation of data trends, abnormal early warning and auxiliary decision-making, as shown in Figure 11. This process provides strong support for building an intelligent monitoring system that integrates collection, transmission, processing and decision-making. Therefore, for various types of sensors, one of the key points of functional expansion lies in the optimization of data transmission mechanisms and the enhancement of platform-based processing capabilities. The selection of transmission mode should be combined with the application scenario and environmental conditions to ensure the reliability and adaptability of the system. For instance, in marine environments, sensors need to interact with autonomous underwater vehicles (AUVs) or buoy systems for information exchange. Due to the complex seabed topography and the difficulty in laying wired media (such as cables and optical fibers), wireless communication methods are more often adopted for marine monitoring. Common underwater wireless communication technologies include three forms: electromagnetic communication, acoustic communication and optical communication. Each of them has its own characteristics and is suitable for different distances, rates and stability requirements [158,159,160]. After the data transmission is completed, the collected data needs to be integrated into a unified early warning platform. Through the visualization module, multi-dimensional real-time display and remote monitoring can be achieved, thereby significantly enhancing the operational transparency and response speed of the system [161]. The data management model of this early warning platform not only helps to monitor trend analysis and fault early warning, but also provides a technical foundation for remote control and multi-sensor collaboration. The stability of the data transmission link and the integration capability of the visualization platform have become core elements that cannot be ignored in the design of intelligent sensing systems. The subsequent content will systematically review the latest developments in wired transmission (such as cable communication and optical fiber communication), wireless transmission (such as acoustic communication, optical communication and electromagnetic communication), and early warning platforms.

### 4.1. Sensor Data Transmission Mode

In monitoring systems for complex environments such as underground, high temperatures, high pressures, and complex liquids, after sensors complete on-site data collection, they need to transmit the data to the monitoring platform in an efficient, stable, and anti-interference manner. This is a key link to ensure the overall system performance and response capability. The selection of data transmission mode not only directly affects the real-time performance and reliability of the system, but also must take into account complex working conditions such as temperature, pressure, medium environment and electromagnetic interference. Therefore, the data transmission mechanism of sensors has become an important component of intelligent monitoring systems in complex environments. For special application scenarios such as underground and underwater, the current mainstream data transmission technologies can mainly be divided into two categories: wired transmission methods and wireless transmission methods. There are significant differences between the two types of transmission schemes in terms of transmission rate, signal stability, environmental adaptability and deployment difficulty. In practical applications, based on the physical characteristics of the monitored environment and system requirements, multiple key indicators such as transmission distance, signal attenuation characteristics, medium interference sensitivity and economic cost should be comprehensively evaluated to select the most suitable communication method, so as to achieve efficient system operation and reliable data acquisition.

To better illustrate the interdependence between sensor devices and data transmission in harsh environments, Table 5 summarizes representative harsh conditions, applicable sensor types, and suitable transmission methods for each environment. It also highlights the collaborative characteristics between sensors and communication technologies based on their respective features. This directly reflects the coupling relationship among environmental constraints, sensor properties, and transmission strategies. Based on this mapping, data transmission methods can be further categorized into wired and wireless approaches. In the following subsection, we first discuss representative technological advances in wired transmission.

#### 4.1.1. Wired Transmission

Wired transmission relies on linear media to achieve data transmission, among which the main forms include power line communication and optical fiber communication.

- Power Line Communication

**Figure 12:**
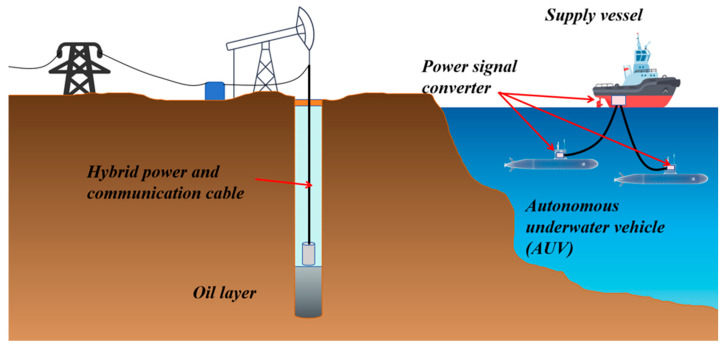
Schematic diagram of power line communication.

In underground working environments such as oil wells, where space is limited and working conditions are complex, the layout of data communication equipment needs to fully consider volume constraints and environmental adaptability. Due to the limited space underground and the existence of electromagnetic interference among various devices, compared with wireless communication, wired communication performs better in terms of anti-interference ability and transmission stability, and has become one of the mainstream choices for underground data transmission at present. Especially on the premise that there is no need to lay additional Communication cables, Power Line Communication (PLC) technology demonstrates good engineering practicability and deployment economy because it integrates communication signals and electrical energy transmission in the same line [162]. This method achieves the modulation and demodulation of carrier signals through single-core cables, providing a low deployment cost and high integration data transmission solution for intelligent underground monitoring systems [163]. However, the high-temperature underground environment poses a severe challenge to the performance of PLC systems. An increase in temperature will cause changes in the electrical parameters of power lines, resulting in power attenuation during transmission and a decline in signal stability. For this purpose, Liangbin Xu et al. designed a high-temperature communication system based on DC PLC (DC-PLC), which can operate stably in an environment of 150 °C. By introducing an adaptive decoding algorithm and optimizing the decoding factor to the adjustable range of the maximum filter, the long-term stable control of the bit error rate (BER) in the duplex communication link was achieved. The experimental results show that the BER fluctuation of this system is very small under long-term high-temperature operation, verifying its communication reliability in extreme underground environments [164]. In addition, PLC technology has also been extended to the communication scenarios of autonomous underwater vehicles (AUVs), aiming to reduce the need for external communication cables and simplify the charging and information exchange processes with supply vessels. A high-bitrate PLC method based on pulse position modulation (PPM) has been proposed, which uses power signal superposition technology to achieve DC power line communication. On this basis, the system can ensure the stability of the bus voltage and the input and output voltages of the power converter during data transmission, with the maximum transmission rate reaching 1 Mbps, and it has good communication, real-time performance and power supply coordination [165]. As shown in Figure 12, it is a basic structural schematic diagram of the power line communication system.

- Optical Fiber Communication

**Figure 13:**
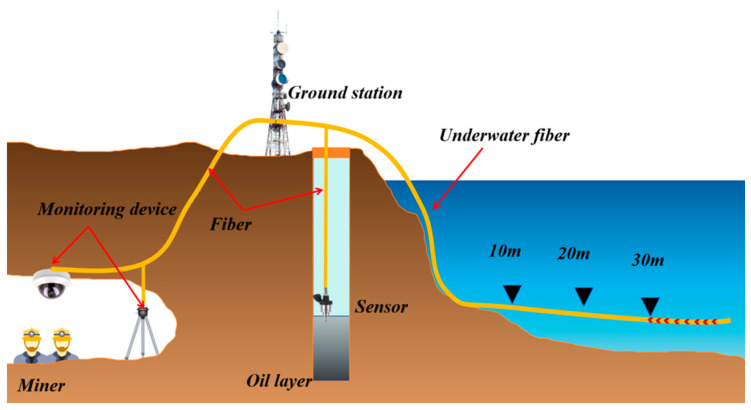
Schematic diagram of optical fiber communication.

Optical fiber communication, as another important wired data transmission method, has demonstrated outstanding performance in monitoring systems in extreme or complex environments. Compared with power line communication (PLC), optical fiber communication has a higher transmission rate, lower latency and stronger anti-electromagnetic interference capability. It is particularly suitable for scenarios such as mines and oil wells, where communication stability and real-time performance are highly demanded. This technology can achieve high-speed data transmission from underground to surface, providing a strong technical guarantee for safety assessment and accident early warning in underground working environments [166,167]. In recent years, the deep integration of optical fiber communication and distributed sensing technology has expanded the application boundaries of optical fiber communication in monitoring systems. For example, in the safety monitoring of oil and gas pipelines and wellbores, an architecture based on optical fiber integrated sensing and communication (ISOC-OF) is proposed, which realizes the synchronous acquisition and transmission of data communication and vibration signals through the same wavelength channel. Under the condition of a bit rate of 56 Gbit/s, this scheme can achieve a transmission performance improvement of approximately 1.3 dB and a transmission power enhancement of 7 dB, providing a new solution for highly integrated intelligent well systems [168]. In the deep-water environment of the ocean, traditional optical fiber communication systems are confronted with the problem of micro-bending loss induced by lateral pressure and high water pressure. When the loss exceeds a certain threshold, it will lead to communication interruption. To address this issue, researchers have developed a new type of trench-assisted optical fiber with a cladding concave profile, which effectively suppresses micro-bending loss through a special structure. Experiments show that the micro-bending loss of the optical fiber prepared at 1500 nm under a weight of 6 kg is only 0.0052 dB/m, which is far superior to that of similar optical fibers. Such as SMF-28e (1.306 dB/m), G.657A1 (0.4252 dB/m), G.657A2 (0.2096 dB/m), and G.657B3 (0.1392 dB/m), significantly improved the communication stability in complex environments [169,170]. In addition, optical fiber communication can also be combined with distributed acoustic sensing systems (DAS) to achieve large-scale and high-resolution monitoring by using existing optical cables. This system converts continuous optical cables into a densely distributed array of strain sensors through an optical demodulator, enabling high-density monitoring of environmental parameters such as underwater temperature changes and wave dynamics. It also directly transmits real-time data to the shorel-based platform, providing a practical technical path for continuous perception of the marine environment [171]. As shown in Figure 13, it is a schematic diagram of the application of optical fiber communication in complex environments.

#### 4.1.2. Wireless Transmission

Wireless transmission technology can significantly simplify system layout, reduce physical connection requirements, effectively enhance space utilization efficiency and system flexibility, and is particularly suitable for scenarios with limited space, frequent environmental dynamic changes, or high difficulty in cable layout. At present, the wireless communication methods widely used in extreme environments mainly include electromagnetic communication, acoustic wave communication and optical communication. Each type of communication method demonstrates specific adaptability and performance advantages in different environments based on its wave propagation characteristics.

- Electromagnetic Communication

**Figure 14:**
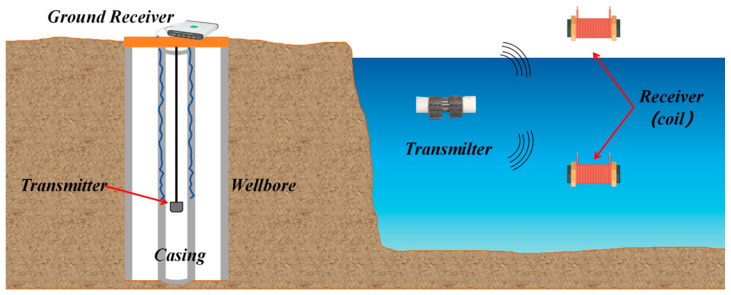
Schematic diagram of electromagnetic communication.

Electromagnetic communication is one of the most widely used and highly accepted wireless communication methods at present, and is widely applied in sensor monitoring systems in various complex environments such as on the ground, underwater and underground. It mainly relies on electromagnetic waves as information carriers to achieve wireless transmission of data over long distances. Compared with acoustic or optical communication methods, the propagation process of electromagnetic waves in air, water and gas-water interfaces is relatively stable, less affected by the Doppler effect, and has higher anti-interference ability and environmental adaptability [172]. In addition, electromagnetic waves have a higher propagation speed and exhibit stronger robustness in the face of factors such as suspended particles and turbidity in seawater [173], providing important support for data transmission in complex medium environments. In underwater monitoring and communication systems, electromagnetic communication, with its ability to penetrate water bodies and a simple communication architecture, has gradually become one of the potential technologies for information exchange between underwater sensor networks (UWSN) and autonomous underwater vehicles (AUVs) [174]. For instance, Silei Yang et al. designed a long-range underwater electromagnetic communication system based on an ultra-compact very low frequency (ELF) magnetic mechanical transmission antenna (UEMTA), and conducted communication experiments underwater and across the gas-water boundary under different modulation methods (SW, STW, and SIW). The system successfully achieved an effective communication rate of 0.1 bps under background noise conditions, with a maximum transmission distance of 210 m, verifying the feasibility and robustness of underwater communication under extremely low frequency conditions [175]. In addition to underwater applications, electromagnetic communication also plays a significant role in the field of underground monitoring. Due to the complex structure and high-temperature and high-pressure environment inside oil wells, the deployment of traditional wired communication is difficult. However, electromagnetic waves have a certain ability to penetrate the formation and casing, providing an effective solution for real-time monitoring of oil wells [176,177]. Studies show that wireless data transmission within the wellbore can be achieved by using extremely low-frequency (ELF, 5–20 Hz) electromagnetic waves. Through finite element simulation and field tests, researchers found that within a depth range not exceeding 1600 m, a signal intensity of more than 0.3 mV could be stably maintained [178]. Although electromagnetic waves have problems such as electromagnetic shielding effect and signal attenuation in the underground environment, resulting in a relatively low overall transmission rate (generally less than 20 bps), it is expected to further improve the transmission performance by increasing the transmission power, optimizing the frequency selection and improving the algorithm at the receiving end. Electromagnetic communication, with its excellent environmental adaptability and strong anti-interference ability, has shown broad application prospects in data transmission under extreme environments. As shown in Figure 14, the electromagnetic communication system has a compact structure and is suitable for embedded integration. It can be used to meet the high-reliability wireless transmission requirements in various engineering scenarios, including underwater and underground.

- Acoustic Communication

**Figure 15:**
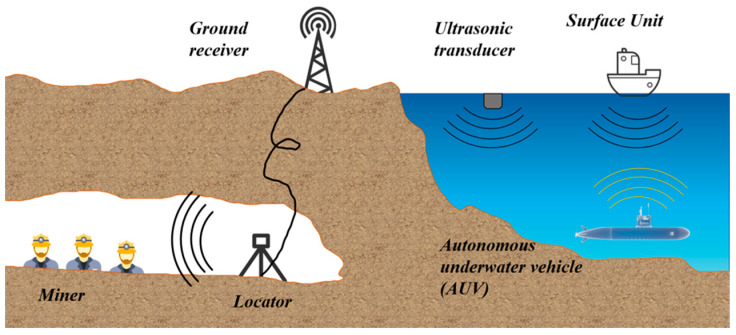
Schematic diagram of acoustic communication.

Acoustic communication is currently the most widely used data transmission technology in underwater environments, especially suitable for medium- and long-distance communication scenarios. It features long transmission distance, low energy loss, and strong resistance to multi-path interference, and has become the mainstream means in the field of underwater communication. In complex marine environments, such as deep water areas or regions with severe terrain obstructions, sound waves have better penetration capabilities than light or electromagnetic waves, making them of significant application value in deep-sea surveying, autonomous underwater vehicle (AUV) positioning, and monitoring of seabed facilities. Due to the lack of visual means in deep-sea environments, the effects of traditional optical or electromagnetic positioning methods are limited. For this reason, research has been conducted on an ultra-short baseline (USBL) transceiver system based on multi-beam forward-looking sonar (MFLS) and integrated acoustic communication modules, achieving real-time positioning and precise docking of underwater vehicles. Experiments have proved that this system can control the average docking error within 0.6 m in deep-water environments, with a docking success rate close to 90%, significantly enhancing the autonomy and guidance accuracy of AUV docking [179]. With the wide application of acoustic communication in underwater scenarios, its communication security has gradually become a research focus. Zhuochen Li et al. proposed a bionic acoustic communication strategy based on quasi-orthogonal keying. This strategy achieves a secure acoustic communication mechanism with high concealment and anti-interference capability by simulating whale whistles for information camouflage and using quasi-orthogonal splicing waveforms for modulation, providing a new solution for high-security demand scenarios such as underwater military communication [180]. In addition to marine environments, acoustic communication also plays a significant role in complex ground environments such as mines. Due to the insufficient light, complex spatial structure and severe attenuation of radio signals inside the mine, the acoustic positioning system has become a key technology to ensure the safety of underground workers and the operation of equipment. This system is typically composed of acoustic transmitters deployed underground and receivers installed on personnel or equipment. It estimates the position coordinates through the time difference in sound wave propagation, thereby achieving real-time positioning and tracking of miners and equipment [180,181,182,183]. This type of acoustic communication system not only enhances the safety of underground operations but also provides technical support for rescue and emergency management. Acoustic communication, with its outstanding medium and long-distance transmission capabilities and adaptability to complex environments, holds an irreplaceable and significant position in underwater and underground communication systems. As shown in Figure 15, the acoustic communication system has a relatively simple structure and is suitable for various complex environments with limited space and an obstructed line of sight.

- Optical Communication

**Figure 16:**
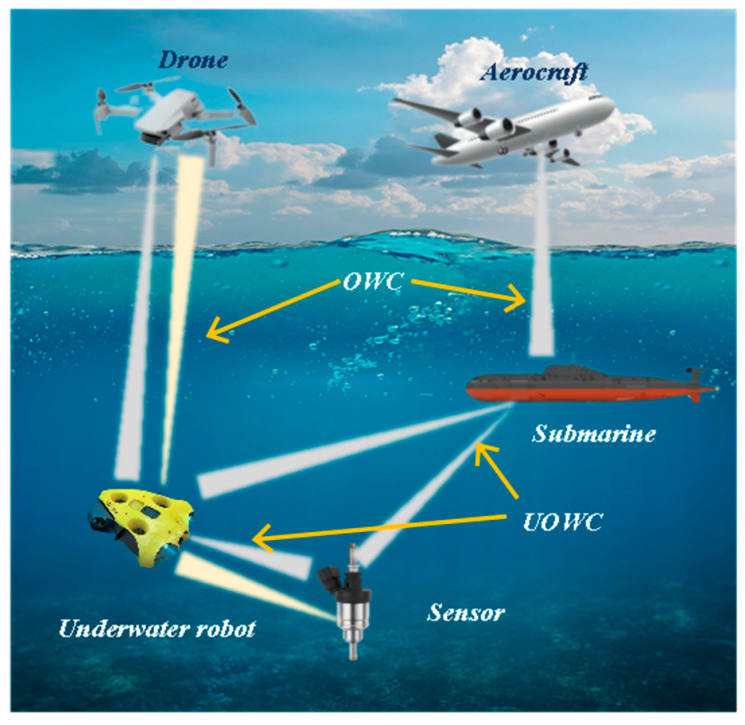
Schematic diagram of optical communication.

Optical communication, as a high-speed and low-latency data transmission method, is taking up an increasing proportion in wireless communication systems, especially demonstrating outstanding performance in underwater environments. Compared with traditional acoustic communication and electromagnetic communication, underwater optical wireless communication (UOWC) has significant advantages such as greater bandwidth, higher data rate and lower latency [184,185]. In addition, water–air optical wireless communication (OWC), as a cross-media communication method, is also becoming a key technical path for achieving real-time data transmission from underwater equipment (such as autonomous underwater vehicles, AUVs) to aerial platforms (such as relay unmanned aerial vehicles or aircraft) [186]. Although optical communication has many advantages in underwater and water–air environments, it still faces many technical challenges in practical applications. Among them, the optical turbulence phenomenon in water bodies has a significant impact on the performance of the UOWC system. Optical turbulence mainly stems from the random fluctuations in temperature and salinity in seawater, thereby causing disturbances in the refractive index of water. This disturbance will cause sharp fluctuations in the light intensity at the receiving end, namely the “Scintillation effect”, which seriously affects the link stability and communication quality [187,188]. By configuring the Beam Collimator at the transmitting end and using the Aperture Averaging Lens at the receiving end, the influence of the spatial disturbance of the beam on the signal can be effectively alleviated, and the Scintillation Index can be significantly reduced. Improve the consistency of signal reception and the stability of communication links [188]. In the water-to-air optical wireless communication link, apart from optical turbulence, sea surface waves and their associated bubble layers also cause non-negligible interference to the light propagation path. The experimental results show that as the sea surface wind speed increases, the bubble density rises accordingly, further intensifying the scattering and absorption of light, causing a significant attenuation of the signal power at the receiving end, thereby reducing the overall transmission efficiency and reliability of the communication system [189,190]. This problem is particularly severe at the interface between the atmosphere and water bodies, posing a challenge to the real-time performance and stability of the OWC link. For the complex disturbance mechanisms of optical communication in marine environments, future research needs to further focus on directions such as turbulence modeling, anti-disturbance structure design, and intelligent link compensation mechanisms, to develop more anti-interference underwater and water-to-air optical wireless communication systems, in order to achieve efficient data transmission in multiple environments. As shown in Figure 16, it is a schematic structure of optical communication in underwater and water–air links.

### 4.2. Engineering Early Warning Platform

In complex underground, downhole oil and gas, and underwater monitoring systems, key performance parameters such as data volume, communication bandwidth, and latency thresholds are interrelated and must be considered simultaneously during system-level design. Specifically, an increase in sensor data volume—driven by higher sampling rates or higher-resolution sensors—directly increases the bandwidth requirements of both wired and wireless transmission links. When available bandwidth is limited, excessive data transmission may lead to increased latency [191,192], potentially exceeding pre-set thresholds for real-time sensing and early warning. System platform performance is constrained by the interplay among these three parameters: data volume determines bandwidth demand [193], bandwidth affects latency [194], and latency thresholds define acceptable limits for data throughput [195]. For example, in underground PLC systems, reliable data transmission up to 1 Mbps under high-temperature conditions can be achieved with adaptive decoding to maintain latency within operational limits [164]. In optical fiber-integrated sensing networks, bit rates on the order of tens of Gbit/s enable low-latency real-time monitoring of structural vibrations and fluid parameters [196]. Meanwhile, wireless acoustic or electromagnetic links demonstrate that limited bandwidth at low frequencies can result in higher latency, constraining effective monitoring range [197]. Therefore, in the design of early warning platforms, careful coordination between sensor resolution, communication bandwidth, and latency requirements is essential to ensure stable and efficient data transmission while maintaining rapid response and reliability of the platform under varying environmental conditions.

The construction of the early warning platform system is based on sensor monitoring modules and data communication systems. It relies on multiple sensors to collect various physical parameters in underground or underwater environments and achieves efficient data transmission through wired or wireless communication methods, thereby building a comprehensive monitoring platform with real-time perception, remote monitoring and intelligent early warning capabilities [198]. At present, a variety of integrated monitoring systems have been developed for underground and underwater applications under complex environmental conditions. Their main application fields cover underground tunnel structure health monitoring, mine environment monitoring and equipment control, multi-parameter monitoring in wells, and marine environmental data collection, etc.

Take tunnel engineering as an example. By installing wireless sensor nodes on the surface of the tunnel structure, continuous monitoring of parameters such as stress, deformation, temperature and humidity can be achieved. When the monitoring value exceeds the preset threshold, the platform can automatically push early warning information to users, thereby effectively enhancing the safety of tunnel operation and disaster response capability [199]. In this field, the data acquisition capability of sensor devices, communication bandwidth, and early-warning latency thresholds are closely interrelated, collectively underpinning the reliability of early warning platforms.

In the field of mine monitoring, the early warning platform system [200] can upload in real time the environmental parameters (such as temperature, pressure, oxygen content, etc.) collected by sensors distributed in various underground areas to the ground system platform [201], achieving dynamic perception and risk assessment of the underground working environment [202], thereby ensuring the life safety of the workers. In addition, by embedding optical fiber sensors into the underground support structure and linking them with the monitoring platform, the stress state endured by each support can be visually displayed. When the monitoring data experiences sharp fluctuations, the system can automatically identify and trigger the early warning mechanism, effectively preventing the occurrence of disastrous events such as mine earthquakes or instability of supports [203]. The bandwidth of optical fiber communication directly affects data transmission latency, which in turn constrains the real-time sensing capability of early warning platforms.

The early warning platform system also plays a key role in the field of intelligent mining. Relying on the deep integration of sensors and control systems, remote control of underground equipment, intelligent path planning and autonomous decision support can be achieved [204], thereby promoting the transformation of mining operations towards “unmanned and intelligent”, and significantly improving operation efficiency and resource utilization rate [205]. The sampling rate of sensors must be matched to the capabilities of the communication link; otherwise, it may compromise the decision-making speed of the system platform and the real-time execution of control commands.

In petroleum engineering, the oil well monitoring system integrates multiple sensors to measure key parameters such as casing pressure, dynamic liquid level, and wellhead temperature. Combined with the downhole sensor network and platform data analysis module, it can achieve real-time assessment of the oil well’s operating status and effectively avoid the risks of equipment failure and production capacity loss [206]. Meanwhile, by introducing intelligent algorithms to filter, reduce noise and model the monitoring data, the data quality and decision-making accuracy can be further improved [207]. An increase in data volume requires the communication link to have sufficient bandwidth; otherwise, latency may compromise real-time response to downhole anomalies.

In terms of water environment monitoring, early warning platforms have also been widely applied. Taking the Poyang Lake Basin as an example, the remote water level monitoring system acquires real-time water level information of the lake through wireless sensor networks and conducts dynamic early warnings, providing important data support for flood risk prevention and control [208].

In the marine field, remote acoustic monitoring systems integrating sonar sensors and underwater communication modules [209], as well as marine operation platforms that support the management of the operational status of autonomous underwater vehicles (AUVs), can all continuously monitor underwater environmental parameters such as temperature, salinity, water flow, and seabed changes. Provide technical support for marine environment assessment and underwater operation decision-making [210]. In acoustic links, limited bandwidth increases latency, thereby restricting monitoring range and early-warning response speed. Consequently, underwater communication performance becomes a critical constraint on the ability of monitoring platforms to achieve fast and reliable responses.

## 5. Discussion

In the harsh engineering environments considered in this study, the development of sensor technologies increasingly exhibits interdisciplinary characteristics. It involves not only in-depth mechanistic research in mechanical, optical, and acoustic domains, but also the integrated application of multiple sensor types. At the same time, data communication links and early-warning platform systems are evolving toward integration, unification, and convergence. However, several limitations remain in current research:

1. Sensor device stability and operational lifespan

Mechanical sensors such as piezoresistive, capacitive, and piezoelectric types, as well as high-frequency acoustic sensors, face material-related limitations in high-temperature and high-pressure environments. Their sensing diaphragms or piezoelectric elements are prone to plastic deformation under prolonged loading, resulting in permanent damage to the sensitive components, leading to decreased sensitivity and baseline drift. High temperatures further accelerate thermal aging and interfacial stress relaxation, reducing sensor lifespan. Similarly, high-frequency acoustic sensors rely on piezoelectric effects or microstructures for monitoring; due to their high-frequency nature, even minor performance changes are amplified. Long-term operation induces aging in internal piezoelectric ceramics and metal electrodes, directly causing drift. These factors affect both the stability and operational lifespan of the sensors. Current research primarily addresses these issues through post-processing and compensation algorithms, which reduce drift under specific conditions. However, compared to improving stability and longevity through the choice and fabrication of sensor materials, such approaches remain limited by environmental constraints.

2. Biofouling and environmental adaptability of devices

In underwater environments, sensors are susceptible to biofouling from algae, microorganisms, and sediment accumulation. When biofilms adhere to sensor surfaces, they alter the surface characteristics of the sensing elements. For mechanical sensors, this can affect stress sensing or vibration amplitude. For optical sensors, biofilms impact refraction and scattering properties, severely degrading performance. Similarly, acoustic sensors may require signals to penetrate the biofilm during acoustic matching, introducing measurement errors. In deep-sea environments, sensors also face high-pressure conditions and corrosive effects, placing higher demands on the corrosion resistance and hermetic sealing of device housings. Current mitigation strategies primarily rely on manual periodic cleaning, which increases operational and maintenance costs and cannot guarantee long-term stability.

3. Electromagnetic compatibility (EMC) issues

In harsh engineering environments, sensor devices often coexist with motors, electronic equipment, and communication stations. In such strong electromagnetic interference (EMI) conditions, sensors may experience output drift and noise. For piezoresistive sensors, EMI can induce currents in Wheatstone bridges, affecting output voltage and causing signal drift. Capacitive sensors are susceptible to parasitic capacitance effects under strong EMI, leading to measurement deviations and reduced sensitivity. High-frequency acoustic sensors may experience charge induction in piezoelectric materials, altering the propagation conditions of surface waves. These sensors are therefore constrained by electromagnetic compatibility (EMC) issues, which must be mitigated through hardware design, filtering algorithms, and monitoring system layout.

4. Packaging and cost

High-performance sensors often rely on sophisticated packaging designs to ensure high-temperature, high-pressure, corrosion resistance, and hermetic sealing capabilities. Piezoresistive, capacitive, and piezoelectric sensors have achieved large-scale applications across various fields, benefiting from relatively mature packaging processes and lower costs. Resonant sensors, however, have complex internal detection structures, resulting in high packaging costs and limited scalability. Acoustic sensors, due to their excellent underwater transmission capabilities, have been commercialized in certain applications, such as hydrophones, widely used for underwater acoustic communication. Optical sensors, particularly those designed for high-temperature environments, have seen research efforts employing metal-ceramic composites, silicon-based materials, and microstructuring techniques to enhance temperature tolerance. Despite these advances, such sensors remain constrained by complex packaging processes and high costs, limiting commercial adoption. Moreover, current studies largely focus on high-temperature performance optimization, with a lack of comprehensive testing under multiple interfering operational conditions.

5. On-site calibration

Sensors operating long-term in harsh environments are often affected by drift, aging, and biofouling, which can cause measurements to deviate from nominal values. Current calibration approaches primarily rely on static calibration in laboratory settings, using standard equipment to measure sensor responses and generate calibration curves. However, in field conditions such as oil and gas wells, deep-sea monitoring platforms, and high-temperature environments, performing shutdown calibrations significantly increases operational and maintenance costs, making these methods difficult to implement. Some studies have proposed data-driven calibration and compensation algorithms for in situ calibration, but these approaches are typically limited to specific sensors and conditions, lacking general applicability.

6. Lack of standardization and certification

The development and application of sensors for harsh environments face gaps in standardization and technical certification. Studies across different sensor technologies and environmental conditions employ varying test conditions, stability evaluation methods, and performance metrics, resulting in a lack of comparability between devices of different types. For example, sensitivity comparisons between devices of different technologies lack unified standards. Although drift and full-scale error have some industry norms (IEEE 1451 [211], ISO 5725 [212]), definitions vary slightly among manufacturers, reflecting the absence of standardized guidelines. In contrast, metrics such as mean time between failures (MTBF) have relatively mature standards and calculation methods (IEC 61709 [213]) with established certification systems; however, significant variations remain across different environmental conditions. Thus, further development of standardized technical evaluation and certification frameworks is required.

## 6. Future Perspectives

In underground high-temperature, high-pressure and complex liquid monitoring environments, sensor technology is increasingly developing towards high sensitivity, multi-parameter integration, long service life and high reliability. Although progress has been made in several aspects, significant technical and application challenges remain. Future prospects for these specific engineering environments can be summarized as follows:

1. At present, mechanical sensors, especially piezoresistive, capacitive, and piezoelectric types, are relatively mature in both fabrication and monitoring applications. Future developments should focus on miniaturization and modularization, enabling integration into underwater robots and autonomous underwater vehicles [214]. For instance, the operational position of an underwater robot can be determined from the pressure and voltage readings of these sensors. Miniaturized and modular sensors will facilitate more convenient deployment in underwater robotics and other equipment.
2. Optical sensors generally exhibit high resistance to temperature and pressure, as well as strong immunity to electromagnetic interference, making them particularly suitable for monitoring equipment conditions in the confined spaces of oil and gas wells. In addition, their excellent compatibility with optical fiber communication enables low-loss transmission of monitoring data. Due to their high sensitivity, optical sensors can detect minute changes in strain, temperature, and pressure, making them well-suited for monitoring pipeline leaks, structural corrosion, and fatigue [215]. These characteristics make optical sensors highly valuable for energy security and industrial safety applications.
3. Acoustic sensors, particularly high-frequency types, offer unique advantages for underwater structural health monitoring and acoustic detection. By optimizing sensor design, sound wave penetration can be enhanced, enabling the detection of more complex interfaces. These sensors are suitable for monitoring the health of underwater support structures on deep-sea and offshore platforms. With the expansion of offshore wind power, high-frequency acoustic sensors can also be applied to monitor the structural integrity of wind turbine foundations, ensuring safe and long-term operation of underwater components [216]. This provides new technical solutions for the operation, maintenance, and intelligent monitoring of underwater parts of renewable energy equipment.

The future development of sensors for harsh environments will not only depend on the performance enhancement of individual sensor devices but also reflect cross-disciplinary and systematic trends, including advances in data transmission and information processing capabilities. The integrated deployment of multiple sensor types, such as mechanical, optical, and acoustic sensors, enables complementary data acquisition, yielding more comprehensive monitoring in challenging conditions. Moreover, such integration allows for mutual data verification, effectively ensuring the accuracy and reliability of measurements. The rapid advancement of artificial intelligence will further support the processing of multi-modal sensor data, allowing monitoring systems to analyze environmental conditions in a timely and precise manner. Concurrently, breakthroughs in novel materials, particularly those suited for underwater applications, will enhance sensor stability. Presently, underwater monitoring equipment is often constrained by seawater corrosion and the high-pressure conditions of the deep sea; the development of pressure- and corrosion-resistant materials will extend sensor service life. Collectively, these trends indicate that future sensor technology will evolve toward modularity, multi-modal integration, and intelligence, thereby providing robust technical support for the long-term, stable operation of comprehensive monitoring systems.

## 7. Conclusions

With the continuous growth in the demand for sensor applications in the engineering field, sensor technology has made breakthroughs in multiple aspects. This paper reviews sensor technologies applicable to various engineering scenarios, covering mechanical, optical and acoustic sensors based on different conduction mechanisms. Considering the harsh conditions such as high temperature, high pressure and corrosion commonly found in engineering environments, these factors significantly affect the stability and accuracy of sensors during long-term monitoring. In response to the above challenges, this paper systematically analyzes the latest research progress in the current field from the perspectives of sensor material selection, structural design, manufacturing process, and performance optimization, and summarizes its latest applications in various engineering fields.

In addition, the transmission and processing methods of sensor monitoring data are the key links to achieve the systematization and intelligence of engineering monitoring. This article further provides an overview of various data transmission technologies suitable for complex environments, covering both wired and wireless methods. It focuses on comparing and analyzing aspects such as transmission stability, data rate, cost control, and deployment convenience, and briefly discusses the construction and application of the engineering early warning platform system.

Although current sensor technology has made remarkable progress at multiple levels, how to further reduce the system cost while ensuring the accuracy and stability of the monitoring system remains an important challenge for future research and engineering applications.

## Figures and Tables

**Table 1:** The latest application of mechanical sensors in harsh environments (RT = Room Temperature).

Mechanism	Temp Range (°C)	Pressure Range	Oil and Gas Wells	Underwater Environments	Function	References
Piezoresistive	RT–150	0–60 MPa	√	-	Pressure	[76]
	25–700	-	-	-	Pressure	[98]
	-	-	-	√	Pressure	[99]
	−75–600	-	-	-	Pressure	[36]
Capacitive	-	100 MPa	-	√	Pressure	[84]
	RT–350	-	-	-	Pressure	[100]
	-	-	-	√	Pressure, liquid level	[74]
	-	-	-	√	Pressure	[101]
Piezoelectric	-	1790 MPa	-	√	Dynamic pressure	[86]
	-	0–0.739 MPa	-	√	Dynamic pressure	[87]
	100–350	-	√	-	Dynamic pressure	[88]
	-	-	-	√	Dynamic pressure	[102]
Resonant	-	120 MPa	-	√	Pressure	[91]
	−40–150	0–120 MPa	√	-	Pressure, temperature	[94]
	-	120 MPa	-	-	Pressure	[103]
	50–175	2–72 MPa	√	√	Pressure	[104]

RT = Room Temperature is at the end of the header.

**Table 2:** The latest application of optical sensors in harsh environments (RT = Room Temperature).

Mechanism	Temp Range (°C)	Pressure Range	Oil and Gas Wells	Underwater Environments	Function	References
Fabry–Perot	-	-	-	√	Salinity	[114]
	RT–800	-	-	-	Pressure	[117]
	-	0–10 MPa	√	√	Pressure	[118]
Fiber Bragg Grating	50–200	0–40 MPa	√	-	Pressure, temperature	[124]
	-	-	√	-	Pressure	[125]
	-	0–30 MPa	√	-	Pressure	[128]
Michelson	20–600	-	-	-	Temperature	[130]
	100–900	-	-	-	Temperature	[131]
	-	115 MPa	-	√	Pressure, Salinity	[133]

**Table 3:** The latest application of acoustic sensors in harsh environments.

Mechanism	Frequency Range	Sensitivity/ Accuracy	Subsurface/ High-Temperature Infrastructure	Underwater Environments	Function	References
Low-frequency	1–80 Hz	−300 nm/Pa	-	√	Earthquake monitoring	[137]
	20–800 Hz	−159.7 dB	-	√	Communication	[140]
	20–200 Hz	−173.8 dB	-	√	Underwater acoustic detection	[150]
	20–500 Hz	−168.5 dB	-	√	Underwater acoustic detection	[141]
	30–80 Hz	−118 dB re 1 rad/μPa	-	√	Underwater acoustic detection	[142]
	100–1000 Hz	−176.3 dB re 1 rad/μPa		√	Underwater acoustic detection	[151]
High-frequency	150 kHz	−173.70 dB	-	√	Ultrasound imaging	[145]
	50 Hz–150 kHz	-	√	-	Damage detection	[152]
	100 Hz–200 kHz	-	√	√	Damage detection	[153]
	40 kHz	-	√	-	Damage detection	[154]
	180 kHz–1 MHz	−177 dB	-	√	Underwater acoustic detection	[148]
	3 MHz–6 MHz	-	-	√	Underwater acoustic detection	[155]
	2.5 MHz	0.05 mm	√	-	High-temperature pipeline health monitoring	[149]
	>20 kHz	−0.01 mm	√	-	High-temperature pipeline health monitoring	[156]

**Table 4:** Performance summary and decision matrix (RT = Room Temperature).

Mechanism		Sensitivity Level	Operating Temperature/ Frequency Range	Anti-Electromagnetic Interference	Stability	Cost/Packaging Difficulty
Mechanical	Piezoresistive	Several mV/kPa to tens of mV/kPa	−75~700 °C	Medium	Medium (Drift influence)	Low, Mature packaging
	Capacitive	Several fF/MPa	RT–350 °C	Medium	Medium	Medium
	Piezoelectric	~1 mV/psi	100–350 °C	Medium	Medium	Medium
	Resonant	27.3–365 Hz/MPa	−40–175 °C	Medium	High (Long-term stability)	High, Complex packaging
Optical	Fabry–Perot	Several μm/MPa to tens of μm/MPa	RT–800 °C	High	High	High
	Fiber Bragg Grating	~50 pm/MPa	50–200 °C	High	High	Medium to high
	Michelson	~100 pm/°C 10^−4^~10^−5^ PSU	20–900 °C	High	High	High
Acoustic	Low-frequency	−159.7 to −173.8 dB(Scalar Channel) −118 to−176.3 dB re 1 rad/μPa(Vector Channel)	<1000 Hz	Medium	Medium	Medium
	High-frequency	−173.70 to −177 dB	>20 kHz	Medium	Low to Medium (Affected by material aging)	Medium to high

**Table 5:** Matching of environmental conditions with sensor types and transmission methods.

Environmental Conditions	Applicable Sensor Types M: Mechanical O: Optical A: Acoustic			Applicable Transmission Method T1: Wired T2: Wireless			Collaborative Characteristics
High-temperature environment (such as downhole high-temperature zones, industrial high-temperature chambers)	M	m_1_	Piezoresistive	T1	t1_1_	Power line communication (suitable for short-distance communication and power delivery)	Optical sensors are highly coupled with optical fiber communication and exhibit good stability under high-temperature conditions. Mechanical sensors require integration with temperature compensation algorithms and rely on optical fiber or power line communication for data transmission.
		m_3_	Piezoelectric				
		m_4_	Resonant				
	O	o_2_	FBG		t1_2_	Optical fiber communication (EMI-resistant, high-temperature tolerant)	
		o_3_	Michelson				
High-pressure environment (such as deep-sea, high-pressure test chamber)	M	m_2_	Capacitive	T1	t1_2_	Optical fiber communication (long-distance, low-loss)	Capacitive and resonant sensors acquire high-pressure signals, and optical fiber communication ensures accurate and high-speed data transmission. Acoustic sensors, combined with acoustic communication, form a natural transmission link.
		m_4_	Resonant				
	O	o_1_	Fabry–Perot	T2	t2_1_	Acoustic communication (suitable for long-range transmission)	
		o_2_	FBG				
		o_3_	Michelson				
	A	a_1_	Low-frequency				
Downhole oil and gas environment (high temperature and high pressure, EMI, confined space)	M	m_1_	Piezoresistive	T1	t1_1_	Power line communication (simultaneous power delivery and data transmission)	Piezoresistive and capacitive sensors are compact, suitable for deployment in confined spaces, and can achieve low-cost installation using power line communication. Optical sensors are highly coupled with optical fiber communication, enabling accurate and stable data transmission in downhole environments with strong electromagnetic interference.
		m_2_	Capacitive				
		m_4_	Resonant				
	O	o_1_	Fabry–Perot		t1_2_	Optical fiber communication	
		o_2_	FBG				
		o_3_	Michelson				
Underwater environment (such as deep-sea monitoring, marine corrosion monitoring)	M	m_2_	Capacitive	T1	t1_2_	Optical fiber communication (long-distance transmission, corrosion-resistant)	Capacitive and piezoelectric sensors are suitable for dynamic pressure monitoring. Optical sensors are highly coupled with optical fiber communication and exhibit corrosion resistance, enabling long-term monitoring and data transmission in submerged environments. The combination of acoustic sensors and acoustic communication provides stable and efficient operation for large-scale underwater environmental sensing.
		m_3_	Piezoelectric				
	O	o_1_	Fabry–Perot	T2	t2_1_	Acoustic communication (long-range)	
		o_2_	FBG				
		o_3_	Michelson				
	A	a_1_	Low-frequency		t2_2_	Optical communication (high-speed transmission in underwater environment)	
		a_2_	High-frequency				

## Data Availability

This study is a review article and no new data were created or analyzed in this study. All data referenced are available from the cited publications.

## Appendix A

To further enhance the systematic nature and traceability of the literature review, two summary tables have been added in the Appendix A Table A1 and Table A2 provide a categorical summary of the final included studies by technical type and application environment.

**Table A1:** A summary table of literature by technical type.

Technology Type	Included Literature
Mechanical sensors	[74,75,76,77,78,79,80,81,82,83,84,85,86,87,88,89,90,91,92,93,94,95,96,97,98,99,100,101,102,103,104]
Optical sensors	[105,106,107,108,109,110,111,112,113,114,115,116,117,118,119,120,121,122,123,124,125,126,127,128,129,130,131,132,133,134]
Acoustic sensors	[135,136,137,138,139,140,141,142,143,144,145,146,147,148,149,150,151,152,153,154,155,156]
Wired transmission	[162,163,164,165,166,167,168,169,170,171]
Wireless transmission	[172,173,174,175,176,177,178,179,180,181,182,183,184,185,186,187,188,189,190]
Engineering early warning platform	[191,192,193,194,195,196,197,198,199,200,201,202,203,204,205,206,207,208,209,210]

**Table A2:** A summary table of the literature by application environment.

Application Environment	Included Literature
Oil and gas well environment	[32,33,60,72,73,75,76,77,78,80,104,118,121,122,124,125,128,164,168,176,177,178,206,207]
Underwater environment	[56,59,74,84,86,87,91,94,99,101,102,114,115,120,129,133,141,143,144,145,146,148,150,151,154,155,165,169,170,171,172,173,174,175,179,180,184,185,186,187,188,189,190,191,192,193,194,197,208,209,210]
Underground environments such as mines and tunnels	[26,27,28,29,31,34,35,57,58,61,79,153,166,167,181,182,183,199,200,201,202,203,204,205]
Other specialized domains, including high-temperature radiation conditions and aerospace applications	[82,83,88,89,92,96,98,100,117,123,130,131,147,196]
